# Optimized Attenuated *Salmonella* Typhimurium Suppressed Tumor Growth and Improved Survival in Mice

**DOI:** 10.3389/fmicb.2021.774490

**Published:** 2021-12-23

**Authors:** Kang Liang, Rui Zhang, Haiyan Luo, Jinlong Zhang, Zhenyuan Tian, Xiaofen Zhang, Yulin Zhang, Md Kaisar Ali, Qingke Kong

**Affiliations:** College of Veterinary Medicine, Southwest University, Chongqing, China

**Keywords:** *Salmonella* Typhimurium, cancer therapy, delivery, endostatin, TRAIL

## Abstract

The gram-negative facultative anaerobic bacteria *Salmonella enterica* serovar Typhimurium (hereafter *S*. Typhimurium) has always been considered as one candidate of anti-tumor agents or vectors for delivering drug molecules. In this study, we compared several widely studied *S*. Typhimurium strains in their anti-tumor properties aiming to screen out the best one for further optimization and use in cancer therapy. In terms of the motility, virulence and anti-tumor efficacy, the three strains 14028, SL1344, and UK-1 were similar and obviously better than LT-2, and UK-1 showed the best phenotypes among them. Therefore, the strain UK-1 (D) was selected for the following studies. Its auxotrophic mutant strain (D1) harboring ∆*aroA* and ∆*purM* mutations was further optimized through the modification of lipid A structure, generating a new strain named D2 with stronger immunostimulatory activity. Finally, the ∆*asd* derivative of D2 was utilized as one live vector to deliver anti-tumor molecules including the angiogenesis inhibitor endostatin and apoptosis inducer TRAIL and the therapeutic and toxic-side effects were evaluated in mouse models of colon carcinoma and melanoma. After intraperitoneal infection, engineered *Salmonella* bacteria equipped with endostatin and/or TRAIL significantly suppressed the tumor growth and prolonged survival of tumor-bearing mice compared to PBS or bacteria carrying the empty plasmid. Consistently, immunohistochemical studies confirmed the colonization of *Salmonella* bacteria and the expression of anti-tumor molecules inside tumor tissue, which were accompanied by the increase of cell apoptosis and suppression of tumor angiogenesis. These results demonstrated that the beneficial anti-tumor efficacy of attenuated *S*. Typhimurium bacteria could be improved through delivery of drug molecules with powerful anti-tumor activities.

## Introduction

Although great advances have been made in cancer treatment and detection, cancer is still one of the leading causes of death worldwide. Conventional cancer treatments often lack specificity and elicit side effects and dose-limiting toxicity. Targeted cancer therapy is urgently needed now. The potential of using microbes, such as bacteria and oncolytic viruses, to fight cancer has been documented for over 100 years (Forbes et al., 2018). Many facultative or obligate anaerobic bacteria, such as *Clostridium* (Malmgren and Flanigan, 1955), *Bifidobacterium* (Kohwi et al., 1978), *Escherichia coli* (Stritzker et al., 2007), and *Salmonella* (Low et al., 1999), have been shown to possess intrinsic tumor-targeting and tumor-killing activities. Especially, the anti-tumor potential of facultative anaerobic *Salmonella* Typhimurium has been extensively studied in the past three decades. Engineered *S.* Typhimurium can directly exert tumor-killing activities or act as a live delivery vector for a wide variety of anti-tumor molecules (Liang et al., 2021).

Attenuated *S.* Typhimurium strains currently used for cancer therapy are mainly derived from wild type strains 14028, SL1344, UK-1, etc. For example, the famous lipid A-modified (*msbB*^−^) and adenine-auxotrophic (*purI*^−^) *S.* Typhimurium strain VNP20009, and the tumor-seeking strain A1-R with defects in the synthesis of arginine and leucine, were both generated from the strain 14028 (Low et al., 1999; Zhao et al., 2006; Hayashi et al., 2009; Hoffman, 2011; Matsumoto et al., 2015). The well-known *aroA* mutant strain SL7207 was the mutagenized derivative of SL1344 (Hoiseth and Stocker, 1981). To maximize the immunostimulatory activity of bacteria for cancer therapy, Felgner et al. and our group have attempted to optimize *S.* Typhimurium strain UK-1 to synthesize homogeneous hexa-acylated lipid A (Felgner et al., 2016b; Liang et al., 2018). Although genome sequences of these wild-type strains from different sources are highly homologous (Luo et al., 2012), bacterial properties may not be the same, such as the virulence and anti-tumor ability. Thus, we aimed to choose one from these strains after multiple comparisons to further optimize the tumor-targeting and delivery of powerful anti-tumor molecules in this study.

It has been widely recognized that angiogenesis, the formation of new capillaries from pre-existing vasculature, is critical for solid tumor growth, invasion, and metastasis (Weidner et al., 1991). Therefore, the suppression of angiogenesis inside tumor tissue has also been considered as one promising strategy for cancer therapy. Because anti-angiogenic therapy targets endothelial cells in the tumor vasculature rather than genetically unstable cancer cells, the probability of developing drug-resistance is reduced, especially, when repeated dosing is needed. Endostatin, the 20-kDa C-terminal fragment of type XVII collagen, is one potent inhibitor of angiogenesis (O’Reilly et al., 1997; Poluzzi et al., 2016). It can bind to a variety of receptors on the surface of endothelial cells with high affinity, thereby blocking the proliferation and migration of endothelial cells and inducing cell apoptosis (Shichiri and Hirata, 2001; Hanai et al., 2002; Walia et al., 2015). For example, endostatin competitively binds to endothelial cells, blocks vascular endothelial growth factor (VEGF)-induced tyrosine phosphorylation of KDR/Flk-1, and thus affects mitogenic activities of VEGF on endothelial cells (Kim et al., 2002). In 2005, Endostar, a novel recombinant human endostatin purified from *E. coli* with an additional his-tag structure, was approved by the State Food and Drug Administration (SFDA) of China for the treatment of non-small cell lung cancer (Yang et al., 2006). Since then, endostatin were increasingly tested in clinical trials for a variety of other different cancers, including malignant serous effusion, melanoma, colon, gastric, breast, and nasopharyngeal cancers, etc. (Li et al., 2018). Besides, in order to exert the therapeutic anti-tumor activity of endostatin effectively, different delivery vectors have been tried including viruses, plasmids, microspheres, and live attenuated bacteria (Li et al., 2010, 2013; Jia et al., 2012; Liang et al., 2018).

Tumor necrosis factor (TNF)-related apoptosis-inducing ligand (TRAIL), also known as Apo-2 ligand (Apo2L), belongs to TNF cytokine superfamily (Daniels et al., 2005). TRAIL is a homotrimeric protein and can induce apoptosis by interacting with its receptors *via* well-elucidated extrinsic and intrinsic apoptotic pathways (Wong et al., 2019). The extrinsic apoptotic pathway is triggered by TRAIL *via* its receptor TRAIL-R1 (DR4) or TRAIL-R2 (DR5), both of which contain a conserved death domain motif (Walczak et al., 1997). It has been uncovered that, in most cancer cell lines, TRAIL-induced apoptosis signal also requires intensification from the activation of the intrinsic apoptotic pathway *via* the B-cell lymphoma 2 (Bcl-2) regulated mitochondrial pathway (Holland, 2013). In addition to DR4 and DR5, TRAIL also interacts with TRAIL-R3 and TRAIL-R4, but they prevent the transmission of apoptosis signals due to the lack or truncation of cytoplasmic death domain (Merino et al., 2006). These “decoy receptors” are widely expressed in normal cells and provide protection against TRAIL-induced apoptosis (Daniels et al., 2005). Thus, the activity of TRAIL to induce apoptosis is specifically targeting toward cancerous cells and sparing normal cells. Since TRAIL receptors DR4 and DR5 are usually overexpressed in different malignant tumors, cancer therapies based on TRAIL or other DR4/DR5 agonists have attracted widespread attention. Different therapeutic agents have been developed to activate the TRAIL apoptosis pathway and tested in many human clinical trials (Stuckey and Shah, 2013; de Miguel et al., 2016; Wong et al., 2019).

Many studies have suggested that one single agent, such as *Salmonella* or one certain drug, is often difficult to elicit sustained anti-tumor effects. Using tumor-targeting *Salmonella* strains for delivery of powerful anti-tumor molecules has been considered as a promising strategy. In this study, we aimed to optimize attenuated *Salmonella* vector to simultaneously deliver the apoptosis inducer TRAIL and angiogenesis inhibitor endostatin, expecting that both cancer cells and proliferating endothelial cells of tumor vasculature could be targeted.

## Materials and Methods

### Cells

The CT26 (mouse colon carcinoma) and B16F10 (mouse melanoma) cell lines were purchased from the Cell Bank of Chinese Academy of Sciences Type Culture Collection. Cells were grown in RPMI 1640 (CT26) or high-glucose DMEM (B16F10) supplemented with 10% fetal bovine serum (FBS) and 1% penicillin–streptomycin and cultured at 37°C in a humidified atmosphere of 5% CO_2_. Human umbilical vein endothelial cell (HUVEC) was purchased from Mingzhoubio (MZ-0746, Ningbo, China) and grown in endothelial cell medium (ECM) supplemented with 5% FBS, 1% penicillin–streptomycin, and 1% endothelial cell growth supplements (ECGS). Cells were counted using a Fuchs-Rosenthal counting chamber and seeded into 96-well plates (1 × 10^5^ cells per well), 24-well plates (2 × 10^5^ cells per well), 6-well plates (5 × 10^5^ cells per well), 25 cm^2^ culture dishes (5 × 10^6^ cells per dish), or 75 cm^2^ culture dishes (2 × 10^7^ cells per dish). The culture medium was changed until cells reached 80% confluence.

### Bacterial Strains and Plasmids

Bacterial strains, plasmids, and primers used in this study are listed in Table 1 and Supplementary Table S1. The suicide plasmid-mediated mutation method was used to introduce precise scarless mutations into *Salmonella* as described previously with minor modifications (Edwards et al., 1998). For the deletion of *purM*, two pairs of primers, D*purM*-1F/D*purM*-1R and D*purM*-2F/D*purM*-2R, were used to amplify upstream and downstream DNA fragments (≈ 400 bp) of *purM* gene, respectively, from the genome of wild type *Salmonella* UK-1. These two fragments were fused *via* their homologous part and inserted into pYA4278 following the protocol of circular polymerase extension cloning (CPEC; Quan and Tian, 2011) to construct the desired suicide plasmid (pSS263). Then, *asd*^−^ *E. coli* χ7213 was transformed with pSS263 by electroporation and grown on LB agar plates containing chloramphenicol (25 μg/ml) and Diaminopimelic acid (DAP; 50 μg/ml). After the conjugation of *E. coli* χ7213 carrying pSS263 and the parent *Salmonella* strain, recombination of suicide plasmids into the chromosome were selected on LB agar plates supplemented with chloramphenicol (negative selection). Screened *Salmonella* clones were grown for 2–4 h until an OD_600_ value of 0.4–0.6 was reached, diluted appropriately, and spotted on NaCl-free plates supplemented with 5% sucrose (positive selection). Colonies were replica patched onto two plates, one of which was supplemented with antibiotics used to select for the suicide plasmid. Those sensitive to the chloramphenicol antibiotic were further purified on sucrose plates and their genotypes were examined by PCR. A similar strategy was used to construct other suicide plasmids and mutant strains (Table 1).

**Table 1:** Strains and plasmids used in this study.

Strains or Plasmids	Descriptions	Sources
**Strains**		
14,028	Wild type S. Typhimurium strain (A)	Roy Curtiss lab
SL1344	Wild type S. Typhimurium strain (B)	Roy Curtiss lab
LT2	Wild type S. Typhimurium strain (C)	Roy Curtiss lab
UK-1	χ3761, wild type S. Typhimurium strain (D)	Lab collection
SL7207	∆aroA mutant of SL1344 (E)	Roy Curtiss lab
A1	∆aroA ∆purM mutant of 14,028 (A)	This study
B1	∆aroA ∆purM mutant of SL1344 (B)	This study
C1	∆aroA ∆purM mutant of LT2 (C)	This study
D1	∆aroA ∆purM mutant of UK-1 (D)	This study
D2	∆pagP ∆pagL ∆lpxR mutant of D1	This study
D2-asd	∆asd mutant of D2	This study
E. coli χ7232	endA1 hsdR17 (rK−, mK+) supE44 thi-1 recA1 gyrA relA1 Δ(lacZYA-argF) U169 λ pir deoR (φ80dlac (lacZ)M15)	Lab collection
E. coli χ7213	thi-1 thr-1 leuB6 glnV44 tonA21 lacY1 recA1 RP4-2-Tc::Mu λ pir ΔasdA4Δzhf-2::Tn10	Lab collection
E. coli χ6097	F- ara ∆(pro-lac) thi Φ80d lacZ ∆M15 rpsL ∆asdA4	Lab collection
**Plasmids**		
pYA4278	SacB mobRP4 R6K ori CmR, pRE112	Lab collection
pSS262	∆aroA suicide plasmids	Lab collection
pSS263	∆purM suicide plasmids	This study
pYA4288	∆pagP suicide plasmids	Lab collection
pYA4284	∆pagL suicide plasmids	Lab collection
pYA4287	∆lpxR suicide plasmids	Lab collection
pSS021	∆asd suicide plasmids	Lab collection
pYA3342	Asd+; pBR ori; Ptrc	Lab collection
pYA4088	Asd+; pBR ori; Ptrc bla ss pspA	Lab collection
pYA3342-ES	Asd+; pBR ori; Ptrc bla ss endostatin	This study
pYA3342-PSMA-ES	Asd+; pBR ori; Ptrc bla ss PSMA scFv-endostatin	This study
pYA3342-RGD4C-ES	Asd+; pBR ori; Ptrc bla ss RGD4C-endostatin	pYA3342-PSMA-ES
pYA3342-RGD10-ES	Asd+; pBR ori; Ptrc bla ss RGD10-endostatin	pYA3342-PSMA-ES
pYA3342-TRAIL	Asd+; pBR ori; araC PBAD lacI; Plac UV5 T7 pol; PT7 TRAIL	This study
pYA3342-TRAIL-ES	Asd+; pBR ori; araC PBAD lacI; Plac UV5 T7 pol; PT7 TRAIL; Ptrc bla ss endostatin	pYA3342-TRAIL
pYA3342-TRAIL-RGD4C-ES	Asd+; pBR ori; araC PBAD lacI; Plac UV5 T7 pol; PT7 TRAIL; Ptrc bla ss RGD4C-endostatin	pYA3342-TRAIL
pYA3342-TRAIL-RGD10-ES	Asd+; pBR ori; araC PBAD lacI; Plac UV5 T7 pol; PT7 TRAIL; Ptrc bla ss RGD10-endostatin	pYA3342-TRAIL
pYA3342-TRAIL- PSMA-ES	Asd+; pBR ori; araC PBAD lacI; Plac UV5 T7 pol; PT7 TRAIL; Ptrc bla ss PSMA scFv-endostatin	pYA3342-TRAIL

The plasmid pYA4088, which encodes *asd* gene and contains *bla* secretion signal sequence under P_trc_ promoter, was used for construction of different expression plasmids. The codon-optimized cDNA of endostatin and its fusion fragments with different targeting peptides (RGD4C, RGD10 and anti-PSMA scFv) were independently cloned into pYA4088 immediately downstream *bla* ss, generating plasmids pYA3342-ES, pYA3342-RGD4C-ES, pYA3342-RGD10-ES, and pYA3342-PSMA-ES. The commercial plasmid PET-28(a) was engineered for the expression of another anti-tumor molecule TRAIL involved in this study. Briefly, we replaced kanamycin resistance gene with *asd* and used the P_BAD_ promoter together with *araC* to regulate the expression of *lacI* from the plasmid. The gene encoding T7 RNA polymerase with its lac UV5 promoter was cloned from BL21(DE3) and inserted into the plasmid. The codon-optimized cDNA of TRAIL was inserted under the control of T7 promoter, generating TRAIL-expression plasmid named as “pYA3342-TRAIL.” For the co-expression of endostatin and TRAIL, open reading frames (ORFs) of endostatin without or with targeting peptides together with P_trc_ promoter and rrnB terminator (T1T2) were, respectively, inserted into pYA3342-TRAIL between *asd* and T7 terminator. Finally, empty pYA3342 and different expression plasmids (*asd*^+^) were transformed into the engineered *Salmonella* bacterial strain, of which *asd* was deleted. In this expression system, P_BAD_ promoter is used to regulate the expression of the repressor LacI. Since the promoter of T7 RNA polymerase, T7 promoter and P_trc_ promoter contain *lac* operator sequence, none of these promoters will be activated when bacteria are grown with the supply of sufficient arabinose. In the absence of arabinose or in *in-vivo* conditions, both T7 promoter and P_trc_ promoter will be activated to initiate the transcription of downstream genes encoding anti-tumor molecules because of no repressor expressed.

### Preparation of *Salmonella* Bacteria for *in vitro* and *in vivo* Experiments

All bacterial strains were cultured on LB agar plates or in LB broth containing appropriate supplements and/or antibiotics. One single clone of bacterial strains was picked, inoculated into LB broth, and grown overnight in a shaking incubator (37°C, 180 rpm). Next day, the overnight culture was diluted 100-fold into fresh medium and grew to exponential phase with an optical density value at 600 nm (OD_600_) of 0.8–0.9. Bacterial cells were then harvested by centrifugation (4,000 × g for 10 min), washed with phosphate buffer saline (PBS), and diluted to obtain desired concentration in an appropriate volume for *in vitro* and *in vivo* experiments. The concentration of bacterial cells was calculated as follows: 0.8 OD_600_ ≈ 1.0 × 10^9^ CFU.

### Determination of Bacterial Phenotypes

The phenotypes of bacterial strains were determined *in vitro*, and all assays were repeated at least twice. The growth of bacterial strains in LB broth (37°C, 180 rpm) was measured every hour, with an initial OD_600_ value of less than 0.05. Bacterial swimming motility was assessed on LB plates solidified with 0.3% agar (wt/vol) as described previously (Kong et al., 2011c). In brief, 5 μl of bacterial suspension (approximately 5 × 10^6^ CFU) was inoculated into the middle of semi-solid plates and subsequently incubated at 37°C for 8 h. The swimming diameter was measured with a ruler. Outer membrane proteins (OMPs) were purified from bacterial strains as described previously (Kang et al., 2002), subjected to sodium dodecyl sulfate-polyacrylamide gel electrophoresis (SDS-PAGE) and stained using coomassie brilliant blue. The lipopolysaccharide (LPS) profile of *Salmonella* bacterial strains was determined by silver staining following the method of Hitchcock and Brown (Hitchcock and Brown, 1983).

### Invasion, Immunofluorescence, and CCK-8 Assays

The invasion assay was performed for testing bacterial infection in cancer cells as described previously (Liang et al., 2018). Cancer cells were seeded into 24-well plates 16 h prior to infection to obtain a density of 5 × 10^5^ cells per well. A total of 5 × 10^7^ CFU of *Salmonella* bacteria prepared as described above was then added to achieve the desired multiplicity of infection (MOI) of 100:1, and the mixture was incubated at 37°C under 5% CO_2_ for 2 h. After washing three times with PBS, cancer cell line-optimal medium containing gentamycin (200 μg/ml; Sigma) was added for additional 1 h of incubation to kill extracellular bacteria. Intracellular bacteria were harvested after washing and extraction with the lysis buffer (0.05% Triton X-100 diluted in PBS). Finally, the diluted lysate was plated onto LB plates and the plates were incubated at 37°C overnight for counting alive bacterial cells (CFU).

The indirect immunofluorescence assay was performed to further confirm the invasion of *Salmonella* bacteria into cancer cells. Cells were seeded into 6-well plates containing coverslips and cultured for 16 h. Prepared bacterial strains were washed twice with PBS, diluted in cell culture medium without antibiotics, and added to cancer cells at a ratio of 100:1. After co-incubation for 2 h and killing of extracellular bacteria by gentamycin, cells were washed gently with PBS and fixed in 4% paraformaldehyde. Then, fixed cells were permeabilized by 0.1% Triton X-100 and incubated with the rabbit anti-*Salmonella* polyclonal antibody (ab156656, Abcam) for 2 h at room temperature (RT) or overnight at 4°C. After washing, the goat anti-rabbit IgG polyclonal antibody conjugated with Alexa Fluor 488 (ab150077, Abcam) was added for 1 h of incubation at RT. Cytopainter phalloidin-iFluor 555 Reagent (ab176756, Abcam) and DAPI (R37606, Invitrogen) was used to indicate cell boundaries and nuclei, respectively. Finally, cell slides were observed and photographed under an inverted fluorescence microscope (Olympus, IX73P2F, Tokyo, Japan).

To test the cell-killing activity of *Salmonella*, cell counting kit-8 (CCK-8) assay was performed. Cells were seeded in 96-well plates and incubated overnight to reach about 80% confluence. After washing twice with PBS, *Salmonella* bacteria resuspended in fresh medium was added for co-incubation with cells at different MOI. After 6 h of incubation, cells were washed gently to remove bacteria. Then, 10 μl of CCK-8 solution (Solarbio, CA1210) per well was added and cells were incubated for additional 2 h. Then, the absorbance was measured at 450 nm with a microplate reader. The cell viability was expressed as the percentage of viable cells compared to the untreated group.

### Western Blotting

The expression of anti-tumor molecules endostatin and/or TRAIL by engineered *Salmonella* bacteria was analyzed by western blotting. When freshly cultured bacteria grew to an OD_600_ value of 1.0, 2 ml of bacterial culture was taken. Bacterial pellets were obtained after centrifugation (12,000 × *g* for 10 min) and proteins present in the supernatant medium were precipitated by trichloroacetic acid (TCA; Sanchez et al., 2009). Bacterial pellets and concentrated supernatant proteins were boiled in 200 μl loading buffer for 10–15 min, and 10 μl of samples were taken for separation by 12% SDS-PAGE gel. Proteins were then transferred to 0.22 μm nitrocellulose membranes using semi-dry electrophoretic transfer cell (Bio-Rad, China). The membranes were probed with mouse anti-endostatin (MA1-40230, Themofisher) or anti-TRAIL monoclonal antibody (ab2219, Abcam), followed by a horseradish peroxidase (HRP)-conjugated anti-mouse IgG secondary antibody (1030-05, Southern Biotech). Immunoreactive proteins were detected using enhanced chemiluminescence (ECL) substrate (BL520A, Biosharp) and visualized by the GelDoc XR+ imaging system (Bio-Rad, China).

### Animals

Seven-week-old female BALB/c and C57BL/6 mice (≈20 g) were purchased from Dashuo Biotechnology Co., Ltd. (Chengdu, China). Animal care, experiments, and euthanasia were performed following the principles stated in the Guide for the Care and Use of Laboratory Animals. All efforts were made to minimize animal suffering during the experiments. All mice were acclimated for 7 days after arrival before experiments started.

The CT26 and B16F10 cell lines were used to establish the colon carcinoma model in BALB/c mice and the melanoma model in C57BL/6 mice, respectively. Briefly, CT26 (5 × 10^5^) or B16F10 cells (2 × 10^5^) suspended in 100 μl of PBS was injected subcutaneously into the right back of each mouse.

### Determination of Bacterial Virulence in Mice

The virulence of *Salmonella* bacterial strains were tested in BALB/c mice as previously described (Kong et al., 2011c). Freshly grown bacteria were harvested, washed, and diluted to the required inoculum density in phosphate-buffered saline containing 0.01% gelatin (BSG). Groups of four mice each were infected orally with various doses of bacterial strains in a volume of 20 μl, ranging from 1 × 10^3^ to 1 × 10^9^ CFU. Then, mice were monitored for 4 weeks post infection and deaths were recorded daily. Experiments were repeated twice. The LD_50_s of different strains were calculated with the software SPSS.

### Colonization of Engineered *Salmonella* Bacteria in Tumor-Bearing Mice

In the colonization experiment, subcutaneous colon carcinoma model was established in BALB/c mice. When tumor volume reached about 200 mm^3^, each mouse was infected intraperitoneally (i.p) with 100 μl of PBS or bacterial suspension containing 5 × 10^6^ CFU (Day 0). Tumor-bearing mice were then euthanized at the indicated dpi (1, 3, 7, 14, and 21) and tissues of spleen, liver, and tumor were taken to determinate bacterial burden. Tissue samples were homogenized and diluted in PBS, and dilutions of 10^1^–10^7^ (depending on the tissues) were plated onto LB plates to count the CFU of viable bacteria. Bacterial burden within normal and tumor tissues was expressed as CFU g^−1^ tissue. The experiment was repeated twice.

### Anti-tumor Effects of *Salmonella* Bacteria

The potential therapeutic anti-tumor effects of different *S.* Typhimurium bacterial strains and recombinant bacteria D2-asd carrying empty or different expression plasmids were evaluated in mouse models of colon carcinoma and melanoma. When tumor masses were visible, tumor-bearing mice were randomly divided into groups (termed as “PBS,” “ST/3342,” “ST/ES,” “ST/TRAIL,” etc.) of eight mice each and injected with 100 μl of bacterial suspension (5 × 10^6^ CFU) or PBS intraperitoneally. Tumor volume (mm^3^) was measured with a caliper every 2–3 days and estimated using the formula (L × W^2^ × π)/6, where L and W represent the length and width of the tumor, respectively. Mice with CT26 tumors exceeding 2,500 mm^3^ were scheduled for euthanasia. For the melanoma model, tumor volume and the survival were recorded until mice died. Meanwhile, body weight of mice was measured as an indicator for general health status. Experiments were performed twice.

### Immunohistochemical (IHC) and H.E Studies

During animal experiments, three mice of each group were euthanatized at 14 dpi, and tumor and normal tissues were taken and fixed immediately with 4% paraformaldehyde for IHC and pathological studies. Standard hematoxylin and eosin (H. E) staining of paraffin-embedded tissues was performed for pathological examination. For the IHC staining, heat-induced antigen retrieval was performed at temperature > 95°C in 10 mM sodium citrate buffer (pH 6.0). Endogenous peroxidase activity was quenched by incubating the sections with 3% hydrogen peroxide for 10 min. After that, the sections were blocked with blocking buffer containing 0.1% Triton X-100, 3% BSA, and 2% normal donkey serum for 1 h, and incubated with following primary antibodies: anti-*Salmonella* (ab156656, Abcam), anti-endostatin (MA1-40230, Themo fisher), anti-TRAIL (ab2219, Abcam), anti-CD34 (ab198395, Abcam), and anti-caspase 3 (ab13847, Abcam; which recognizes a cleaved form of caspase-3 produced after apoptosis has been induced). After washing with PBS, HRP-conjugated secondary antibodies were added. Then, the sections were stained with a freshly prepared 3, 3′-diaminobenzidine (DAB) chromogen and counterstained with hematoxylin. Photos were taken in five random fields of each sample. The integrated optical density (IOD) of positive staining was analyzed by the software Image-Pro Plus 6.0.

### Statistical Analysis

Numerical data were expressed as means ± SEM if not stated. One-way or two-way ANOVA analysis followed by Tukey’s multiple comparisons test was used to evaluate the difference significance of bacterial motility and invasion, cell viability, tumor volume, tumor and spleen weight, and the mean IOD of IHC staining among groups. The Kaplan–Meier survival curve was used for monitoring mouse survival and difference significance was analyzed by the log-rank test. Data analysis was performed using GraphPad Prism 8.0. *p* < 0.05 was considered as statistically significant (*, #, or †); *p* < 0.01 as very significant (**, ##, or ††); and *p* < 0.001 as extremely significant (***, ###, or †††).

## Results

### The *in vitro* Phenotypes of Different *S.* Typhimurium Bacterial Strains

**Figure 1:**
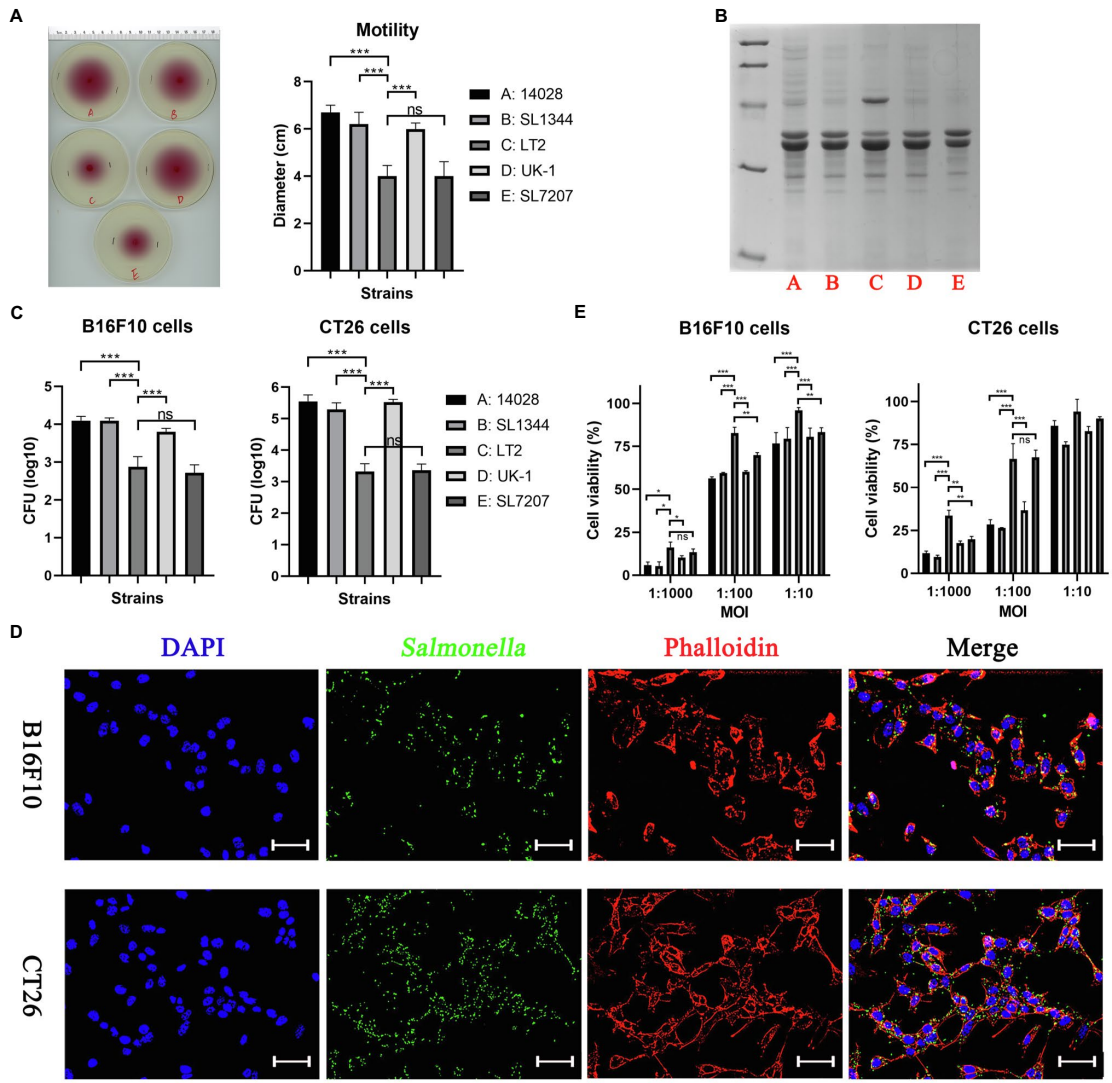
The *in vitro* phenotypes of different *Salmonella* Typhimurium strains. **(A)** Swimming motility of bacterial strains was tested on 0.3% semi-solid agar plates. The swimming diameter was measured with a ruler. **(B)** Outer membrane proteins purified from different *Salmonella* strains were stained by coomassie brilliant blue. **(C)** The ability of bacterial strains to invade cancer cells was determined by counting alive bacterial cells (CFU). Briefly, cancer cells were infected by *Salmonella* for 2 h at an MOI of 100:1, and further cultured with medium containing gentamycin for 1 h to kill extracellular bacteria. **(D)** The invasion of *Salmonella* into cancer cells was also confirmed by the immunofluorescence assay. For visualization by fluorescence microscopy, *Salmonella* bacteria (green) were stained using rabbit anti-*Salmonella* primary antibody and anti-rabbit secondary antibody conjugated with Alexa Fluor 488, followed by cell nuclei (blue) and actin (red) stained by DAPI and phalloidin, respectively. Scale bar, 50 μm. **(E)** The toxicity of bacteria to cancer cells after 6 h of co-incubation was assessed by the CCK-8 assay. The significance of differences among groups were analyzed by two-way ANOVA analysis followed by Tukey’s multiple comparisons test and indicated by asterisks (*p < 0.05; **p < 0.01; and ***p < 0.001).

In this study, *S.* Typhimurium strains 14028, SL1344, LT-2, UK-1, and SL7207 were indicated by A, B, C, D and E for short, respectively. First, we compared different *S.* Typhimurium bacterial strains in their *in vitro* phenotypes. The growth of these bacterial strains was tested in LB broth (37°C, 180 rpm) and no obvious differences were observed (Supplementary Figure S1). All strains grew to the stationary phase after 10 h, showing similar OD_600_ values (≈1.5) of their cultures. Surprisingly, the strain LT-2 (C) showed decreased motility by about one-third when compared to other wild type strains, which was even weaker than the auxotrophic strain SL7207 (E), as indicated by the swimming diameter of bacteria on semi-solid agar plates (Figure 1A). OMPs purified from these strains were stained on SDS-PAGE gel using coomassie brilliant blue and similar bands were shown except that one more protein of about 60 kDa was present in LT-2 (or this protein was expressed in higher abundance; Figure 1B). There were no obvious differences among the LPS profile of these strains detected by silver staining (Supplementary Figure S1B). The interaction between *Salmonella* bacteria and cancer cells of CT26 and B16F10 cell lines was also imitated *in vitro*. It was shown that the strains 14028, SL1344, and UK-1 had comparable ability to invade cancer cells while the invasion of LT-2 was significantly weaker than the above three (Figure 1C). The immunofluorescence assay further confirmed the invasion of *Salmonella* bacteria into cancer cells (Figure 1D). Consistently, LT-2 showed the lowest toxicity to cancer cells and other wild-type strains decreased the viability of cancer cells to the similar extent (Figure 1E).

### The Virulence, Colonization, and Anti-tumor Efficacy of Different *S.* Typhimurium Bacterial Strains in Mice

The virulence of different bacterial strains was tested in BALB/c mice (Table 2). Consistent with previous studies, the virulence of LT-2, which is a major strain for cellular and molecular biology research in *Salmonella* (Swords et al., 1997) is low with the oral LD_50_ of about 1.0 × 10^7^ CFU, which was at least 100 times those of other three wild type strains (A, B, and D; Luo et al., 2012). Besides, the oral LD_50_ of the auxotrophic strain SL7207 (E) was larger than 1.0 × 10^9^ CFU, indicating that mutation of *aroA* was effective in attenuation of bacterial virulence.

**Table 2:** The virulence of different *S.* Typhimurium bacterial strains in BALB/c mice.

Groups	Routes	Doses: death
A: 14028	Orally	1 × 10^5^ CFU/20 μl: 4/4
		1 × 10^4^ CFU/20 μl: 1/4
		1 × 10^3^ CFU/20 μl: 0/4
B: SL1344	Orally	1 × 10^5^ CFU/20 μl: 4/4
		1 × 10^4^ CFU/20 μl: 1/4
		1 × 10^3^ CFU/20 μl: 0/4
C: LT2	Orally	1 × 10^8^ CFU/20 μl: 4/4
		1 × 10^7^ CFU/20 μl: 2/4
		1 × 10^6^ CFU/20 μl: 1/4
		1 × 10^5^ CFU/20 μl: 0/4
D: UK-1	Orally	1 × 10^5^ CFU/20 μl: 4/4
		1 × 10^4^ CFU/20 μl: 2/4
		1 × 10^3^ CFU/20 μl: 0/4
E: SL7207	Orally	1 × 10^9^ CFU/20 μl: 0/4
		1 × 10^8^ CFU/20 μl: 0/4

**Figure 2:**
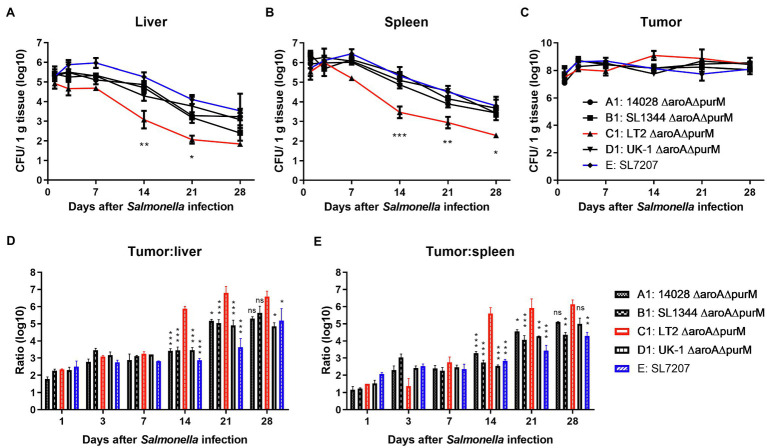
The *in vivo* colonization of different *S.* Typhimurium auxotrophic strains in tumor-bearing mice. To determinate the *in vivo* colonization of different *S.* Typhimurium auxotrophic strains in tumor-bearing mice, four tumor-bearing mice were euthanized at each indicated dpi, and tumor and normal tissues were taken sterilely. Bacterial burdens in the liver **(A)**, spleen **(B)**, and tumor tissues **(C)** were measured by plating serial dilutions of tissue homogenates and expressed as CFU g-1 tissue. **(D,E)** Tumor-to-normal tissue ratios at different dpi were calculated. At dpi 14, 21, and 28, the significance of the difference between the colonization number of C1 and other strains was analyzed. The colonization profiles of C1 and other strains were compared and difference significance was analyzed through two-way ANOVA analysis followed by Tukey’s multiple comparisons test (*p < 0.05; **p < 0.01; and ***p < 0.001).

Colonization experiments were conducted to test the tumor-targeting ability of *Salmonella* bacteria in the subcutaneous model of colon carcinoma. For the attenuation of bacterial virulence, we introduced the ∆*aroA* mutation into different wild type strains, respectively, and generated mutants auxotrophic for aromatic amino acids (AAA) including tyrosine, phenylalanine, and tryptophan. It was also reported that the deletion of *aroA* increased the immunostimulatory capacity of *Salmonella*, an important part of its inherent anti-tumor activity (Felgner et al., 2016a). In addition, the gene *purM* responsible for purine synthesis was also deleted to avoid the possibility of virulence recovery. Finally, constructed ∆*aroA* ∆*purM* mutants (A1, B1, C1, and D1) could grow in LB medium but could not grow in minimal salt medium without the supply of AAA and adenine (Supplementary Figure S2A). On the 1st, 3rd, 7th, 14th, and 21st day after tumor-bearing mice were intraperitoneally injected with *Salmonella* mutants, tissues including tumor, liver, and spleen were picked up to determine bacterial burden. As shown in Figure 2, at dpi 1, 1 × 10^5^ and 1× 10^6^ CFU/g of *Salmonella* bacteria were detected in liver and spleen tissues, respectively, and about 1 × 10^8^ CFU/g in the tumor. Then, the number of colonized bacteria was gradually reduced in normal tissues but remained relatively stable in the tumor (Figures 2A–C). At least within 4 weeks after infection, all these auxotrophic strains preferentially accumulated in the tumor relative to normal tissues, with tumor-to-normal tissue ratios ranging from 100: 1 to over 100,000: 1 (Figures 2D,E). Especially, C1 (LT-2-∆*aroA* ∆*purM*) was cleared from normal tissues more quickly than other auxotrophic mutants including A1, B1, D1, and E, showing significantly lower number of colonized bacteria 2 weeks post infection (Figures 2A,B).

**Figure 3:**
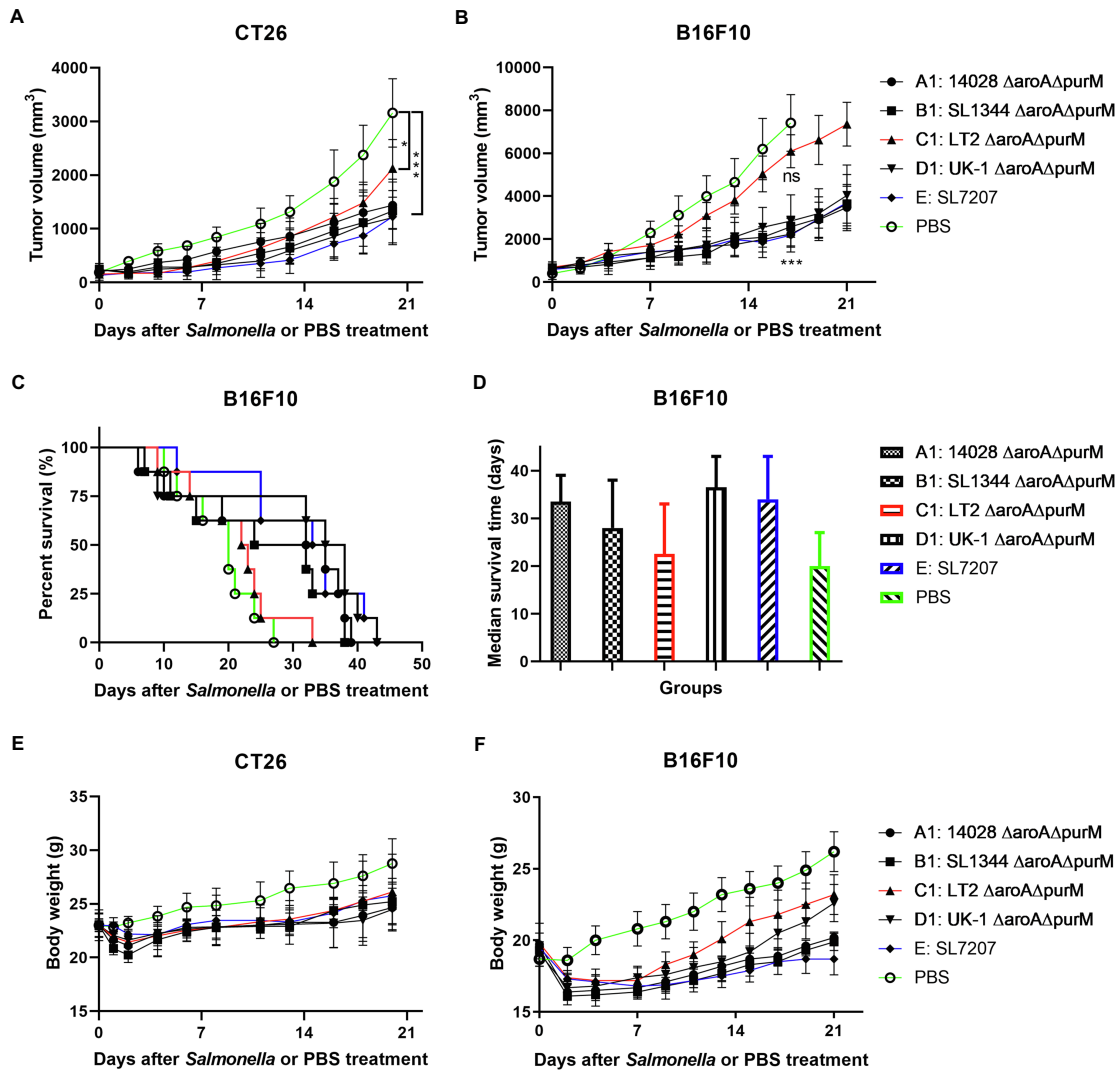
The anti-tumor efficacy of different *S.* Typhimurium auxotrophic strains in mouse models. Tumor-bearing mice were randomly divided into groups of eight mice each and intraperitoneally injected with 100 μl of PBS or different *S.* Typhimurium auxotrophic strains (5 × 10^6^ CFU). Tumor volume of CT26 colon carcinoma- **(A)** and B16F10 melanoma-bearing mice **(B)** were measured every 2–3 days and difference significance between PBS and other groups was analyzed through two-way ANOVA analysis followed by Tukey’s multiple comparisons test (*p < 0.05; **p < 0.01; and ***p < 0.001). **(C)** The Kaplan–Meier survival curves for mice bearing B16F10 melanoma were monitored. **(D)** The median survival time was also analyzed and shown as the median with upper and lower limits. **(E,F)** During animal experiments, body weight of tumor-bearing mice was measured as an indicator for general health status.

We next evaluated the native anti-tumor efficacy of these auxotrophic strains in mice (Figure 3). It was shown that auxotrophic strains of 14028, SL1344, and UK-1 (A1, B1, and D1) possessed similar anti-tumor ability, which is significantly stronger than LT-2-∆*aroA* ∆*purM* (C1) in terms of inhibition of tumor growth (*p* < 0.5 or 0.01). Moreover, 14028-∆*aroA* ∆*purM* (A1), UK-1-∆*aroA* ∆*purM* (D1) and SL7207 (E) obviously improved the survival of melenoma-bearing mice when compared to PBS treatment (33.5, 36.5 and 34, vs. 20 days). The median survival time of C1-treated mice was about 22 days, which was almost the same as PBS group.

Considering the importance of bacterial motility, invasion ability, virulence, and native anti-tumor activity to *Salmonella*-mediated cancer therapy, we finally chose UK-1 from four wild-type strains mentioned above for further studies.

### Optimization of *Salmonella* Bacteria and Expression of Endostatin and/or TRAIL

In this study, we attempted to utilize live attenuated *Salmonella* bacteria to deliver therapeutic anti-tumor agents. Therefore, we further optimized selected auxotrophic strain D1 (UK-1-∆*aroA* ∆*purM*) through introduction of the three mutations *∆pagP ∆pagL ∆lpxR* and generated a new mutant strain named D2 (UK-1-∆*aroA* ∆*purM ∆pagP ∆pagL ∆lpxR*). D2 was expected to synthesize homogeneous hexa-acylated lipid A, which has been considered to possess the most potent immunomodulatory activity, and to reverse the immunosuppression in the tumor microenvironment (Teghanemt et al., 2005; Liang et al., 2019). The phenotypes of auxotrophic strain D1 and its derived lipid A-modified strain D2 were identified together with their wild-type strain UK-1 (D; Supplementary Figures S2, S3). It showed that ∆*aroA* and ∆*purM* mutations resulted in the inability of *Salmonella* to synthesize AAA and adenine (Supplementary Figure S2A) and that generated auxotrophic strains D1 and D2 grew more slowly than the wild-type strain UK-1 in LB broth (Supplementary Figure S2B). The motility of D1 and D2 was decreased as well, according to their swimming on semi-solid agar plates (5.0, 5.0, vs. 6.0 cm; both *p* < 0.05; Supplementary Figure S3A). Related gene mutations did not seem to obviously influence the OMP profile of *Salmonella* bacteria (Supplementary Figure S3B). The LPS staining indicated that the structure modification of lipid A was achieved by *∆pagP ∆pagL ∆lpxR* mutations as well. As shown, the homogeneous ladder of LPS synthesized by the strain D2 was obviously different from other strains (Supplementary Figure S3C). The ability of auxotrophic strains (D1 and D2) to invade and to kill cancer cells was both significantly weaker than D (Supplementary Figures S3D,E). Compared to the wild type strain, D2 was significantly attenuated in mice. After oral administration to mice, D2 was avirulent even at a dose higher than 1.0 × 10^9^ CFU. Upon intraperitoneal infection, the LD_50_ of D2 was more than 1.0 × 10^7^ CFU, while that of UK-1 was only about 1.0 × 10^2^ CFU (Table 3).

**Table 3:** The virulence of UK-1 derived auxotrophic and lipid-A modified strain (D2) in BALB/c mice.

Groups	Routes	Doses: death
D: UK-1	Intraperitoneally	1 × 10^4^ CFU/100 μl: 4/4
		1 × 10^3^ CFU/100 μl: 4/4
		1 × 10^2^ CFU/100 μl: 2/4
D2: UK1-∆*aroA*∆*purM* Δ*pagP*Δ*pagL*Δ*lpxR*	Intraperitoneally	1 × 10^8^ CFU/100 μl: 3/4
		1 × 10^7^ CFU/100 μl: 1/4
		1 × 10^6^ CFU/100 μl: 0/4
D: UK-1	Orally	1 × 10^5^ CFU/20 μl: 4/4
		1 × 10^4^ CFU/20 μl: 2/4
		1 × 10^3^ CFU/20 μl: 0/4
D2: UK1-∆*aroA*∆*purM* Δ*pagP*Δ*pagL*Δ*lpxR*	Orally	1 × 10^9^ CFU/20 μl: 0/4
		1 × 10^8^ CFU/20 μl: 0/4

**Figure 4:**
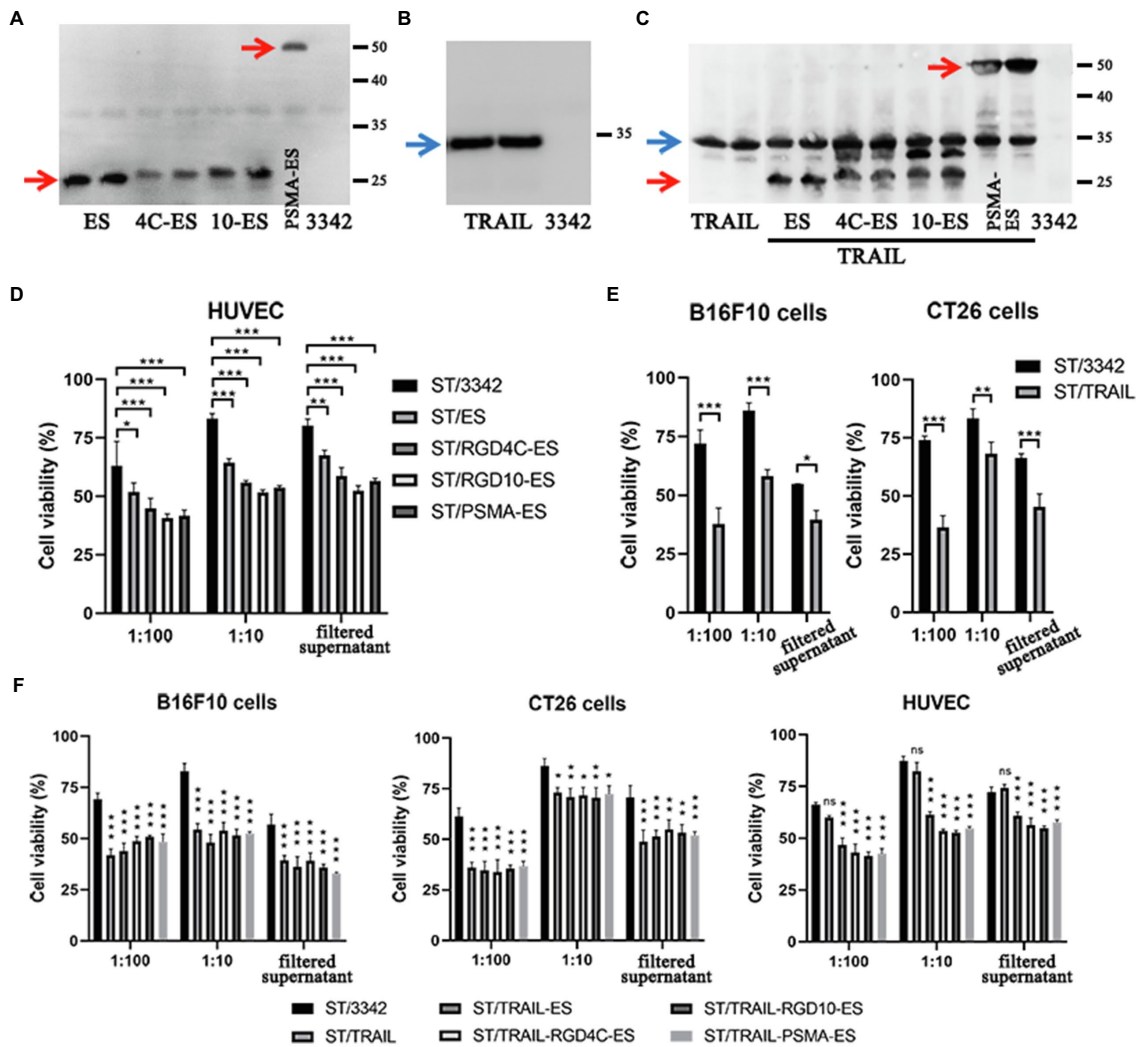
The expression of endostatin and/or TRAIL by optimized *Salmonella* and their *in vitro* cell-killing activities. **(A,B)** The expression of endostatin (ES, RGD4C-ES, RGD10-ES, and PSMA-ES; 25.2, 28.1, 28.4, and 52.6 kDa) and TRAIL (34.3 kDa) by engineered *Salmonella* strain D2-asd was detected by western blotting, respectively. The samples tested were prepared from bacterial lysates. **(C)** Recombinant *Salmonella* co-expressing endostatin and TRAIL were also constructed and verified in the same way. **(D–F)** The toxicity of recombinant *Salmonella* bacteria expressing endostatin and/or TRAIL to HUVECs and cancer cells at the MOI of 1:100 or 1:10 were tested by the CCK-8 assay. Besides, the filtered medium supernatant of bacterial culture containing secreted anti-tumor molecules was also added to cells for testing. The cell viability shown (%) was relative to that of untreated cells, followed by comparison among groups by two-way ANOVA analysis. The cell viability shown (%) was relative to that of untreated cells. Difference significance between ST/3342 and other groups was analyzed through two-way ANOVA analysis followed by Tukey’s multiple comparisons test (*p < 0.05; **p < 0.01; and ***p < 0.001).

Then, we constructed several plasmids (*asd*^**+**^) for expressing anti-tumor molecules including potent angiogenesis inhibitor endostatin (ES) and/or apoptosis inducer TRAIL (Table 1), and transformed them and the empty plasmid, respectively, into the ∆*asd* derivative of D2 (D2-asd, named as “ST” for short when plasmids were carried), where plasmids and bacterial strains constitute a balanced-lethal system to ensure the plasmid stability (Wang et al., 2013). In this study, the β-lactamase signal sequence (bla ss) was added to the N-terminus of anti-tumor molecules for the secretion of them by *Salmonella* bacteria. Besides, to increase the specificity of endostatin’s anti-angiogenic anti-tumor activity, endostatin was linked to short RGD (arginine-glycine-aspartate) peptides or anti-prostate specific membrane antigen (PSMA) single-chain variable fragment (scFv), respectively, both of which could bind to overexpressed surface proteins of proliferating endothelial cells with high affinity and selectivity, i.e., the αvβ3 integrin and PSMA (Liu et al., 1997; Weis and Cheresh, 2011; Danhier et al., 2012; Afshar-Oromieh et al., 2016). The expression and secretion of ES and TRAIL by *Salmonella* bacteria was confirmed by western blotting (Figures 4A–C; Supplementary Figure S4C). Subsequently, the toxicity of recombinant *Salmonella* to the cancer cells or HUVECs was detected by the CCK-8 assay. ES-expressing *Salmonella* was shown to cause lower viability of HUVECs when compared with bacteria carrying the empty plasmid, so did their filtered culture medium (Figure 4D). *Salmonella* expressing TRAIL exerted higher toxicity to cancer cells of CT26 and B16F10 cell lines (Figure 4E). Moreover, when treated with *Salmonella* carrying ES-TRAIL co-expression plasmids, both HUVECs and cancer cells could be killed (Figure 4F).

### The *in vivo* Anti-tumor Effects of Engineered *Salmonella* Bacteria Expressing Anti-tumor Molecules

**Figure 5:**
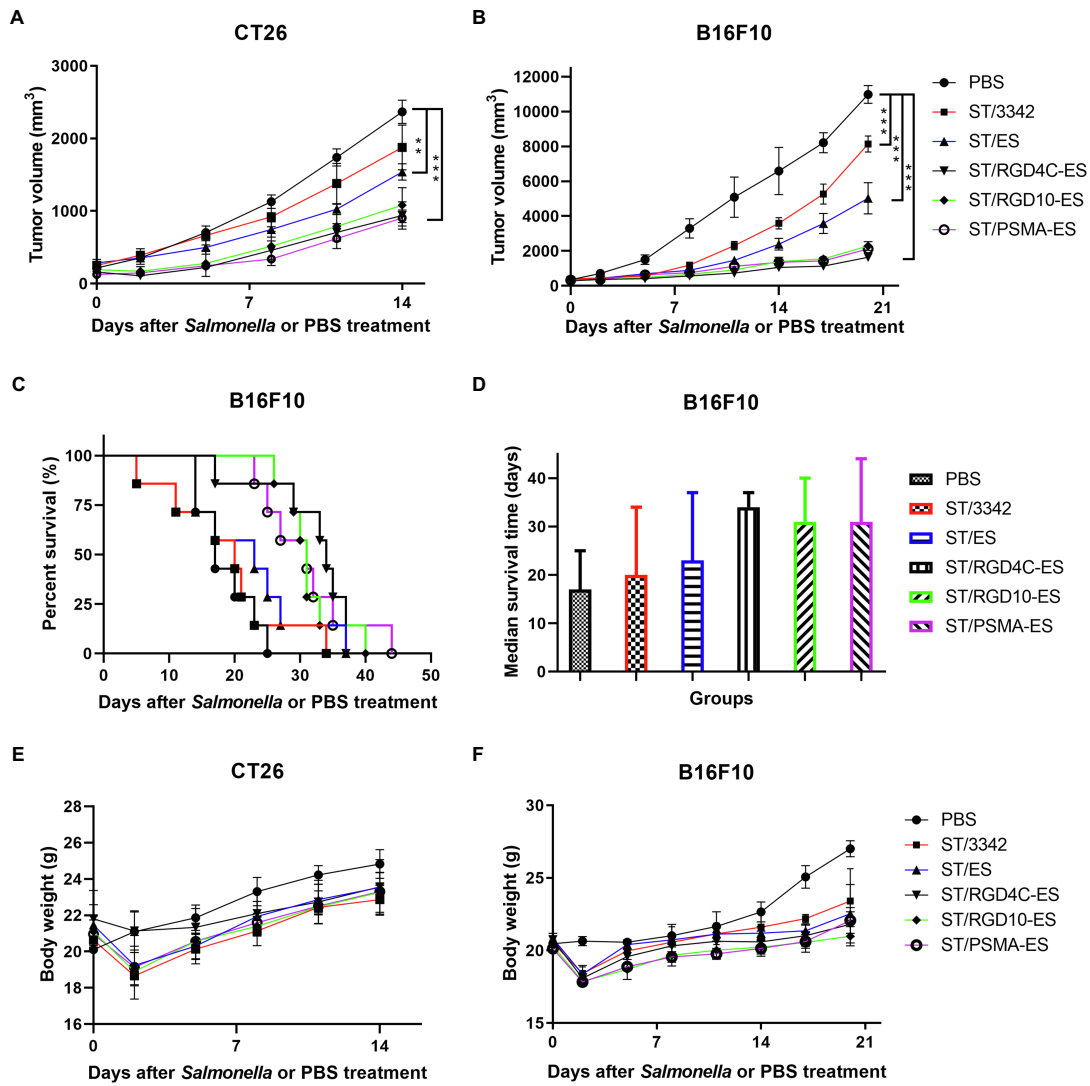
The *in vivo* anti-tumor efficacy of engineered *Salmonella* expressing endostatin. Tumor-bearing mice were randomly divided into groups of eight mice each and intraperitoneally injected with 100 μl of PBS or 5 × 10^6^ CFU of attenuated *Salmonella* (D2-asd) carrying pYA3342 or plasmids expressing ES, RGD4C-ES, RGD10-ES, and PSMA-ES, respectively. **(A,B)** Tumor volume of mice from different groups were measured every 2–3 days and difference significance between PBS and other groups was subjected to multiple comparisons through two-way ANOVA analysis (*p < 0.05; **p < 0.01; and ***p < 0.001). **(C)** for mice bearing melanoma were monitored and the median survival time **(D)** was shown as the median with upper and lower limits. **(E,F)** Meanwhile, body weight of tumor-bearing mice was recorded.

Animal experiments were performed to study the potential therapeutic effects elicited by our engineered *Salmonella* bacteria (D2-asd) and its delivered anti-tumor molecules (ES and TRAIL) mentioned above. When tumor masses were visible, tumor-bearing mice were grouped randomly and intraperitoneally received 5 × 10^6^ CFU of ST/3342, ST/ES, ST/RGD4C-ES, ST/RGD10-ES or ST/PSMA-ES, or 100 μl of PBS. In the CT26 colon carcinoma model, infection of *Salmonella* bacteria carrying different ES expression plasmids significantly delayed tumor growth compared to the treatment with PBS (Figure 5A). At 14 dpi, the mean tumor volume of PBS group was about 2,400 mm^3^, whereas those of other groups were about 1900 mm^3^ (ST/3342, ns, vs. PBS group), 1,500 mm^3^ (ST/ES, *p* < 0.01), 900 mm^3^ (ST/RGD4C-ES, *p* < 0.001), 1,100 mm^3^ (ST/RGD10-ES, *p* < 0.001), and 900 mm^3^ (ST/PSMA-ES, *p* < 0.001), respectively (Figure 5A). Engineered attenuated *Salmonella* carrying expression plasmids of endostatin linked with RGD peptides or anti-PSMA scFv (ST/RGD4C-ES, RGD10-ES, and PSMA-ES) showed significantly stronger suppressive effects on tumor growth compared to ST/3342 (*p* < 0.05 or 0.01, data not shown). These recombinant strains seemed to cause stronger suppression of tumor growth than ST/ES, although the differences were not significant statistically. Tumor tissue was also weighed after mice were sacrificed. The comparison of tumor weight change among different groups was similar with that of tumor volume suppression (Supplementary Figure S5A).

Then, we established the aggressive B16F10 melanoma model to further evaluate the anti-tumor efficacy of our engineered *Salmonella* bacteria. Similarly, mice infected by *Salmonella* carrying ES expression plasmids showed significantly superior therapeutic benefits compared to those treated with PBS or ST/3342 at dpi 14, 17, and 20 (*p* < 0.001; Figure 5B). After 2 weeks, the mean tumor volume of PBS group was about 6,600 mm^3^, whereas those of *Salmonella* infection groups were about 3,600 mm^3^ (ST/3342), 2,400 mm^3^ (ST/ES), 1,100 mm^3^ (ST/RGD4C-ES), 1,300 mm^3^ (ST/RGD10-ES), and 1,300 mm^3^ (ST/PSMA-ES), respectively. Moreover, the life of melanoma-bearing mice was significantly prolonged by treatment with *Salmonella* expressing endostatin linked with different targeting peptides, as shown in Figure 5. In detail, tumor-bearing mice treated with D2-asd carrying RGD4C-ES, RGD10-ES, or PSMA-ES survived for over 30 days, followed by mice treated with ST/ES for 23 days, whereas the median survival time of ST/3342- and PBS-treated mice was 20 and 17 days, respectively (Figure 5D).

**Figure 6:**
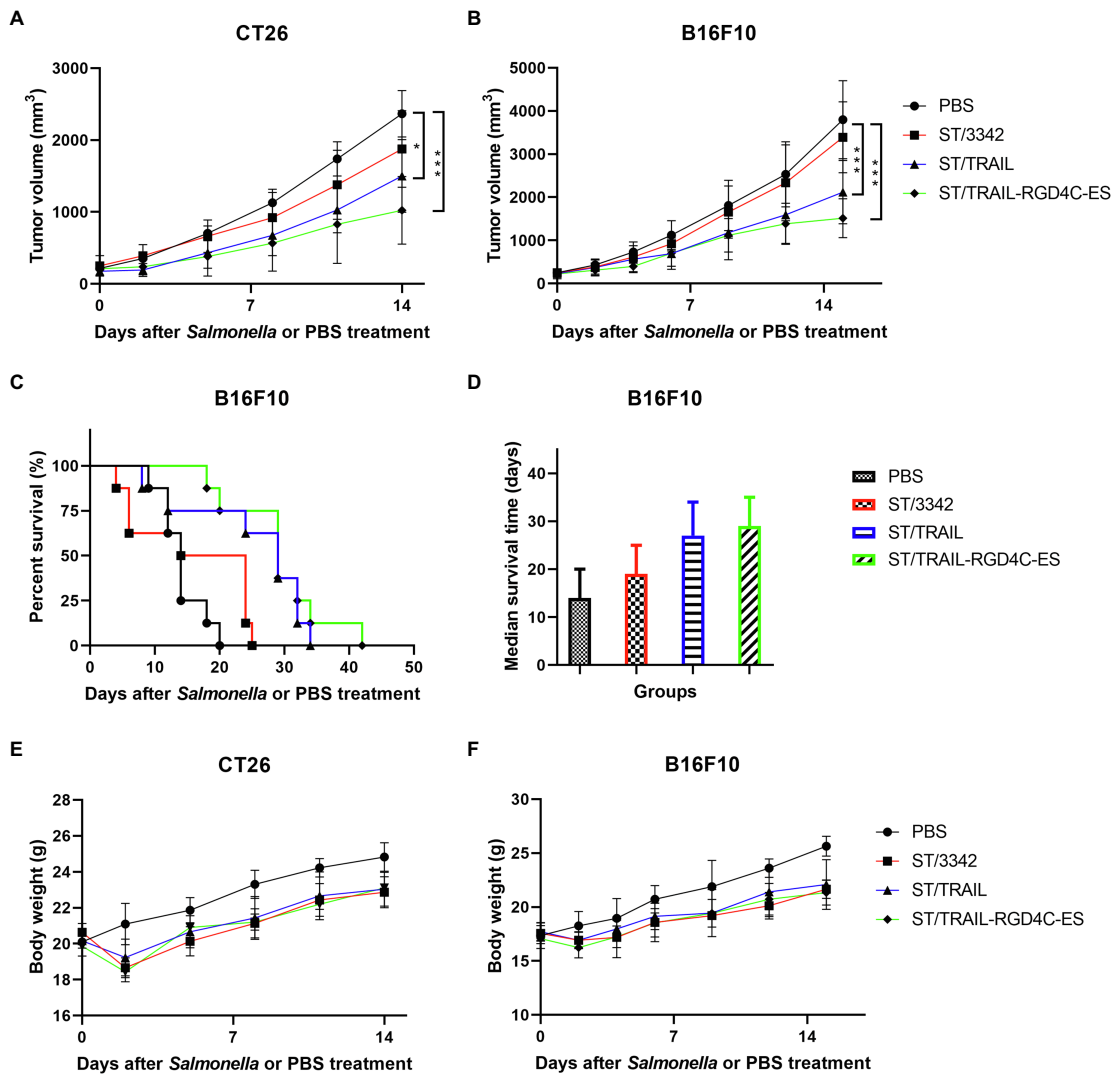
The *in vivo* anti-tumor efficacy of engineered *Salmonella* bacteria expressing TRAIL and endostatin. Tumor-bearing mice were randomly divided into four groups and intraperitoneally injected with 100 μl of PBS or 5 × 10^6^ CFU of attenuated *Salmonella* bacteria (D2-asd) carrying empty pYA3342, TRAIL-expression and TRAIL-RGD4C-ES co-expression plasmids, respectively. **(A,B)** The change of tumor volume were recorded every 2–3 days and difference significance between PBS and other groups was analyzed through two-way ANOVA analysis followed by Tukey’s multiple comparisons test (*p < 0.05; **p < 0.01; and ***p < 0.001). **(C)** The Kaplan–Meier survival curves for mice bearing melanoma were monitored and difference significance was analyzed by the log-rank test. **(D)** The median survival time of mice in different groups was shown as the median with upper and lower limits. **(E,F)** Body weight of tumor-bearing mice was also recorded during animal experiments.

Previous studies have shown that one single anti-tumor agent may not be enough to cause sustained tumor suppression. Targeting different signal pathways may synergistically produce stronger anti-tumor effects. Therefore, we attempted to use the optimized *Salmonella* vector to deliver the apoptosis inducer TRAIL, of which the anti-tumor efficacy was also tested in this study (Supplementary Figures S4A,B), in addition to anti-angiogenic endostatin and expected that both cancer cells and proliferating endothelial cells of tumor vasculature could be targeted. The co-expression plasmid of TRAIL and endostatin linked with RGD4C peptide was constructed and transformed into the strain D2-asd. The anti-tumor efficacy of newly generated recombinant *Salmonella* strain ST/TRAIL-RGD4C-ES was then evaluated in mouse models of colon carcinoma and melanoma. The results showed that significant inhibition of tumor growth and prolongation of mouse survival were caused by treatment of ST/TRAIL-RGD4C-ES or ST/TRAIL, compared to PBS and ST/3342 (Figures 6A–D; Supplementary Figure S5C). Surprisingly, significantly different anti-tumor effects were not observed between ST/TRAIL-RGD4C-ES and ST/TRAIL. The mean tumor volume of melanoma-bearing mice treated with PBS or ST/3342 was more than 3,000 mm^3^, while those of ST/TRAIL and ST/TRAIL-RGD4C-ES treated mice were only 2,100 mm^3^ and 1,500 mm^3^, respectively. The median survival time of mice was increased to 19, 27, and 29 days, respectively, by treatment with *Salmonella* carrying empty, TRAIL-expressing and TRAIL and RGD4C-ES co-expression plasmids, compared to that of PBS group (14 days).

### The Anti-tumor Mechanisms and Adverse Side Effects of Recombinant *Salmonella*

**Figure 7:**
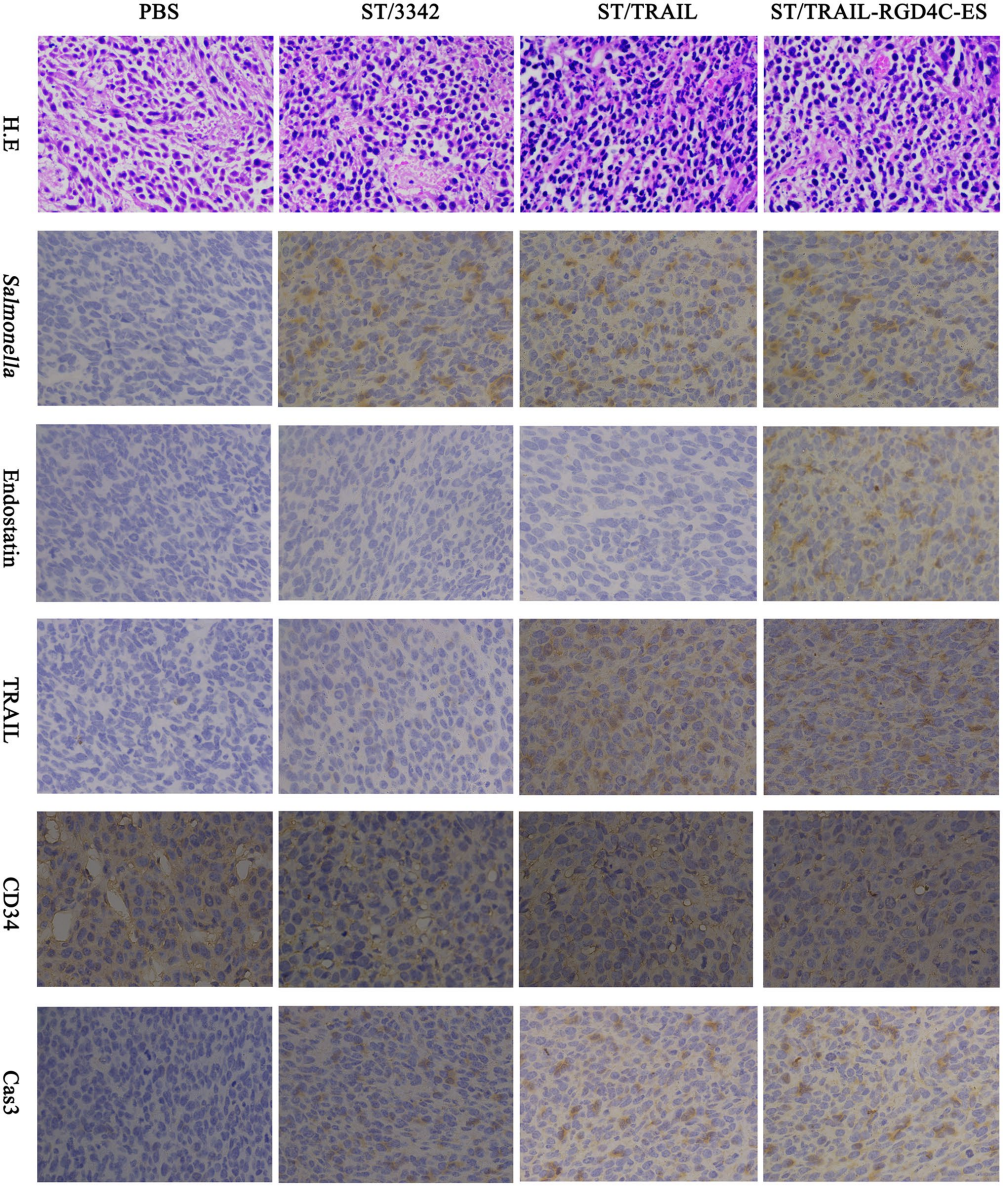
Immunohistochemical studies on tumor tissue. Two weeks after tumor-bearing mice received different treatments, tumor samples were taken for immunohistochemical studies as described above. The accumulation of *Salmonella* bacteria and the expression of anti-tumor molecules inside tumor tissue were detected using specific primary antibodies, respectively, against *Salmonella*, endostatin and TRAIL. Tumor microvascular density (MVD) and cell apoptosis level were also evaluated by staining for CD34 and cleaved caspase-3, respectively. Scale bar, 50 μm.

During animal experiments, tissue samples were also taken for studying the *in vivo* anti-tumor mechanisms and adverse toxic-side effects of our engineered *Salmonella* through IHC and H.E staining. Consistent with the results of colonization experiments, attenuated *Salmonella* carrying empty or different expression plasmids could accumulate inside tumor tissue after systemic infection, which was indicated by staining for *Salmonella* O-antigen. The anti-tumor molecules released by *Salmonella* were also detected there (Figure 7; Supplementary Figures S6, S7).

Since we aimed to utilize optimized live *Salmonella* to mediate cancer therapy combining suppression of angiogenesis and induction of cell apoptosis, *Salmonella* was equipped with plasmid-encoded endostatin and/or TRAIL. To test whether these two molecules and *Salmonella* itself exerted their anti-tumor activities, staining was conducted on tumor tissue for CD34 and cleaved caspase-3, two indicators, respectively, for angiogenesis and cell apoptosis (Fina et al., 1990; Porter and Jänicke, 1999). Compared with ST/3342 and PBS treatment groups, the ST/TRAIL and ST/TRAIL-RGD4C-ES groups showed significantly higher level of cell apoptosis within tumor tissue, which was indicated by the increase of activated caspase-3 (Figure 7; Supplementary Figure S7B). The expression level of CD34, a surface marker of proliferating vascular endothelial cells, was significantly decreased inside tumor tissue infected by ST/TRAIL-RGD4C-ES compared to other treatment groups (Figure 7; Supplementary Figure S7B). Similar results were also observed when mice were infected by *Salmonella* expressing different forms of endostatin (Supplementary Figures S6, S7A). In detail, bacterial delivery of endostatin linked with RGD or PSMA scFv targeting peptides was shown to significantly decrease CD34 expression within the tumor, compared to treatment with PBS or *Salmonella* only. Besides, it showed that *Salmonella* possessed native anti-tumor activities targeting angiogenesis and cancer cells even though it carried the empty plasmid. Taken together, these results demonstrated that the accumulation of *Salmonella* within tumor tissue and local release of TRAIL and endostatin led to apoptosis induction and angiogenesis inhibition, which at least partly explained the superior therapeutic benefits elicited by *Salmonella* co-expressing TRAIL and RGD4C-ES.

**Figure 8:**
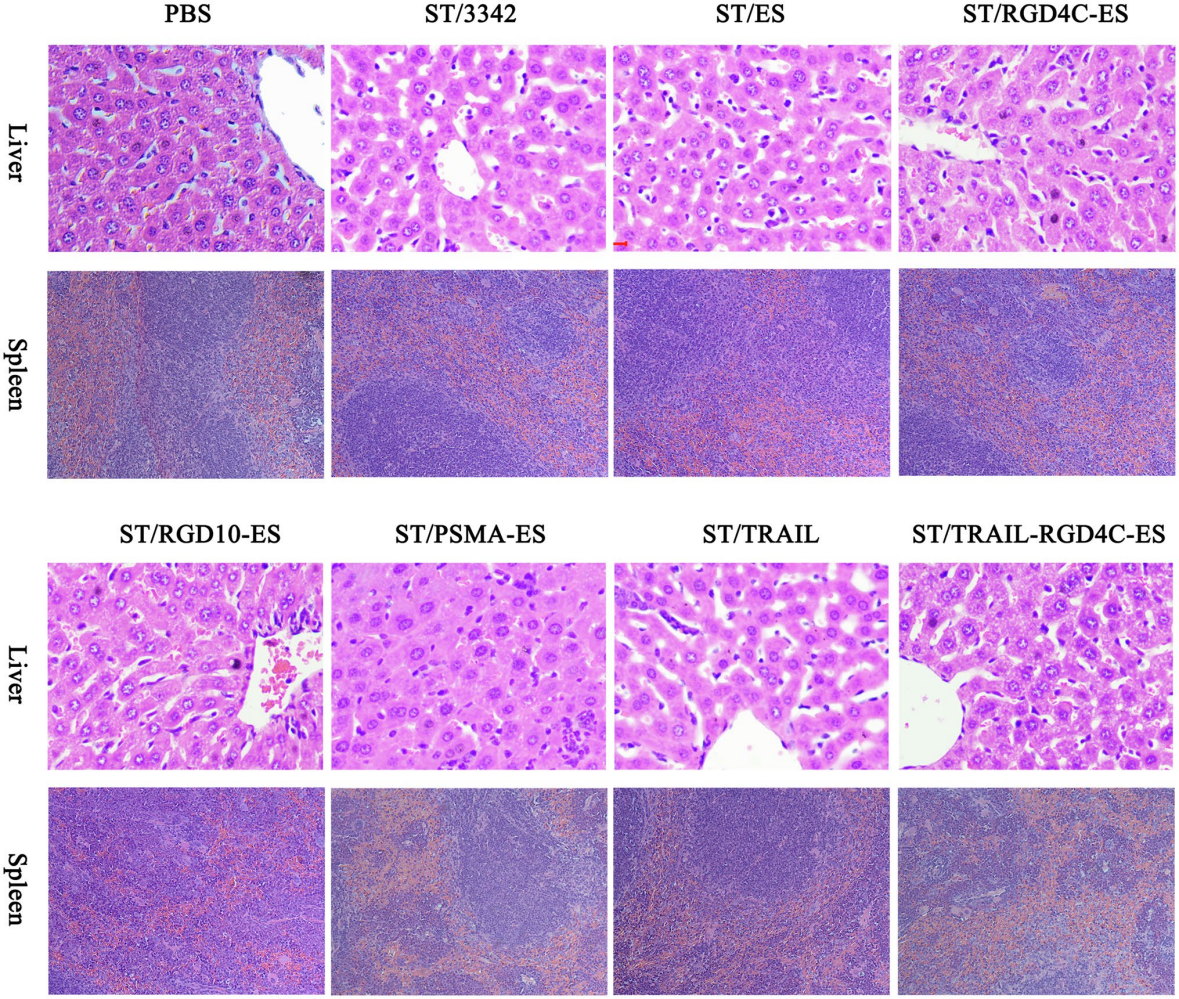
Pathological studies on normal tissues. Standard H.E staining was performed on the liver and spleen tissues taken during animal experiments. In *Salmonella* infection groups, disorderly arrangement of hepatic cords accompanied by swelling and degeneration of hepatocytes was observed in the liver, and the spleen appeared slight inflammation and the infiltration of phagocytic cells and inflammatory cells. There were no obvious pathological changes in PBS treatment group.

During animal experiments, we also monitored the general health of tumor-bearing mice through recording their body weight. The body weight of mice was obviously decreased by about 10–15% in the initial several days after i.p infection of *Salmonella* and gradually recovered 1 week later (Figures 3, 5, 6). It seems plausible that our engineered attenuated *Salmonella* (i.e., D2-asd carrying different *asd*^+^ plasmids) indeed elicited toxic-side effects on tumor-bearing mice at the early infection stage, while the administration dose adopted in this study (5 × 10^6^ CFU for each mouse) was tolerable for mice. Meanwhile, we also took susceptible normal tissues including liver and spleen for pathological studies. The swelling and degeneration of hepatocytes in the liver and the inflammation in the spleen were observed after H.E staining, indicating that i.p infection of *Salmonella* bacteria caused pathological changes on these tissues (Figure 8). Besides, splenomegaly was obvious in mice infected by *Salmonella* (Supplementary Figures S5B,D).

## Discussion

*S.* Typhimurium bacteria have been extensively studied as a candidate anti-tumor agent as well as delivery vector. However, different laboratories have not necessarily chosen the same bacterial strain for in-depth research. Currently, attenuated *S.* Typhimurium derived from the wild type strains 14028 and SL1344, and the highly virulent strain UK-1 were tested for cancer therapy increasingly. Besides, LT-2 is the principal strain for cellular and molecular biology research of *Salmonella* bacteria. Thus, we aimed to test whether their properties are obviously different and to screen out one strain with superior anti-tumor capacity.

*Salmonella*-mediated tumor suppression is not only owing to its intrinsic tumor-killing activity but also to the host immune responses triggered by the adjuvant activity of bacteria, both of which are usually associated with bacterial virulence. For example, *Salmonella* may induce cancer cell apoptosis through nutrient competition, release of bacterial toxins, and down-regulation of the AKT/mTOR pathway (Lee et al., 2014). The host anti-tumor responses can be induced after *Salmonella* infection by recruiting immune cells, such as neutrophils, macrophages, dendritic cells and CD8^+^ T cells, and cytokines including IL-1β and TNF-α into tumor tissue (Kim et al., 2015). In this study, the strain LT-2 showed significantly weaker invasion and toxicity to cancer cells compared with 14028, SL1344 and UK-1 (Figures 1C,E). Moreover, when different *S.* Typhimurium bacterial strains were compared in their virulence in mice, the oral LD_50_ of LT-2 was 100 times higher than those of other three wild-type strains (Table 2). The motility is another potential advantage of live attenuated bacteria that enables treatment of deep tumor tissue (Kasinskas and Forbes, 2007; Toley and Forbes, 2012). It was assumed that bacteria as well as many macromolecular chemotherapeutics enter tumor tissue *via* the openings of 200–2000 nm between disorganized vascular endothelial cells. *Salmonella* may use passive and/or active mechanisms to complete this process. Thereafter, the distribution of chemotherapeutics within tumor tissue depends on passive diffusion and drug concentration drops with distance from the vasculature. Whereas bacteria as complex living organisms are able to acquire energy from surrounding environment and their penetration inside tumor tissue is not entropically limited. According to the *in vitro* observation, the motility of LT-2 was also significantly weaker than that of 14028, SL1344, and UK-1 (Figure 1A). Most importantly, animal experiments demonstrated that attenuated strains with the same mutations of 14028, SL1344, and UK-1 possessed similar anti-tumor efficacy, which was significantly stronger than LT-2 derived attenuated strain in terms of inhibition of tumor growth and improvement of mouse survival (Figures 3A–D). Therefore, we believed that LT-2 is not a good choice for genetically engineering bacteria for cancer therapy. UK-1 (UK stands for universal killer), a phage type 1 strain, is a chicken-passaged isolate of a highly virulent *S.* Typhimurium strain originally retrieved from an infected horse in 1991 (Curtiss and Hassan, 1996). UK-1 is not only highly invasive and virulent for chickens and mice but also is capable of lethal infections in calves, pigs, and horses (Luo et al., 2012). We previously have completed the sequencing and analysis of the whole genome of UK-1. Even though comparison of several frequently studied *S.* Typhimurium strains reveals high collinearity, UK-1 exhibits the highest virulence (Luo et al., 2012). UK-1 has been extensively used in our laboratory for virulence and colonization studies in mice. Because of the high virulence of UK-1, attenuated derivatives of UK-1 are expected to mediate cancer therapy better than those of less virulent *S.* Typhimurium strains. Therefore, UK-1 was chosen for further studies for cancer therapy.

Safety is always a prerequisite for the treatment of diseases including cancer. *S.* Typhimurium with intrinsically pathogenic properties may cause serious toxicity to the host especially after systemic infection. Thus, attenuation of bacterial virulence is necessary for its use in cancer therapy. One commonly considered strategy is introduction of auxotrophic mutations into *Salmonella* bacteria. Theoretically, the adaptability of auxotrophic bacterial strains will obviously decrease in normal tissues but remain unchanged inside the tumor. For example, adenine- (VNP20009) and AAA-deficient *S.* Typhimurium strains (SL7207) were shown to have reduced virulence and high tumor-targeting specificity (Hoiseth and Stocker, 1981; Low et al., 1999). In this study, our engineered auxotrophic strain D1 (UK-1-∆*aroA* ∆*purM*) as well as the ∆*aroA* ∆*purM* mutants of 14028 and SL1344 also preferentially colonized the tumor and was gradually eliminated from normal tissues. Besides, LPS is an important virulence factor for *Salmonella* and other gram-negative bacteria. Lipid A, also called endotoxin, represents the most conserved component of LPS and is responsible for the immunostimulatory and toxic activities of LPS. It has been shown that toll-like receptor 4 (TLR-4) of host cells recognizes extracellular lipid A (Miyake, 2004) and caspase-11 senses intracellular lipid A, thereby stimulating the innate immune responses (Hagar et al., 2013; Kayagaki et al., 2013). Studies of the structure–activity relationship of lipid A indicate that the number, length, and symmetry of acyl chains in lipid A govern its stimulatory activity. Lipid A derivatives containing fewer acyl chains (e.g., penta- and tetra-acylated lipid A) have shown to antagonize or poorly activate TLR-4 and caspase-11 signal pathways (Teghanemt et al., 2005; Matsuura et al., 2012; Ohto et al., 2012). This may partially explain the failure of VNP20009 in human clinical trials (Toso, 2002), which predominantly synthesizes penta-acylated lipid A due to the mutation of *msbB*. Thus, we did not consider this modification in this study. Through multiple mutations, such as Δ*pagP* Δ*pagL* Δ*lpxR*, it is not difficult to construct mutant strains harboring hexa-acylated lipid A with potent immunostimulatory activities (Teghanemt et al., 2005). Here, we generated an attenuated *Salmonella* strain named D2 (UK-1-∆*aroA* ∆*purM* Δ*pagP* Δ*pagL* Δ*lpxR*), which was auxotrophic for adenine and aromatic amino acids, and synthesized hexa-acylated lipid A. As expected, the virulence of D2 in mice was highly reduced compared to its wild type strain UK-1 (Table 3). Besides adequate attenuation, *Salmonella* for cancer therapy should be able to effectively migrate toward, colonize and penetrate inside tumor tissue, which is associated with the motility of bacteria indicated by an *in vitro* model of continuously perfused tumor tissue (Kasinskas and Forbes, 2007; Toley and Forbes, 2012). The *in vitro* swimming assay revealed that the motility of D2 as well as its parent strain D1 was decreased by about one-third compared to that of wild type strain UK-1 (Supplementary Figure S3A). However, the decrease of motility did not seem to compromise the *in vivo* colonization or penetration of *Salmonella* in the tumor (Figure 7; Supplementary Figure S6). D2 was still able to invade and influence the viability of cancer cells *in vitro* although not as effectively as the wild-type strain did (Supplementary Figures S3D,E).

Many previous studies have shown that one single anti-tumor agent, such as one certain drug or attenuated *Salmonella*, often cause incomplete tumor suppression. Treatments targeting different pro-tumor and/or anti-tumor pathways may elicit superior beneficial therapeutic effects. Thus, in this study, the optimized attenuated *Salmonella* strain was used as a live vector to deliver endostatin and TRAIL simultaneously. It was expected that proliferating endothelial cells of tumor vasculature and cancer cells could be targeted, respectively, by these two anti-tumor molecules, thus leading to both suppression of angiogenesis and induction of cancer cell apoptosis. Besides, different short targeting peptides were linked to endostatin, respectively, to increase the specificity of endostatin’s anti-angiogenic activity. Two different tumor models were established in mice to evaluate the potential tumor suppression effects elicited by our engineered *Salmonella* and its delivered anti-tumor molecules. As a result, recombinant *Salmonella* expressing different forms of endostatin (i.e., ES, RGD4C-ES, RGD10-ES, and PSMA-ES) could significantly inhibit tumor growth and improve the survival of mice bearing melanoma. When TRAIL was delivered by *Salmonella*, potent anti-tumor effects were elicited on mice. Moreover, mice treated with *Salmonella* co-expressing TRAIL and endostatin linked with the short RGD4C peptide showed superior therapeutic benefits compared to mice infected by *Salmonella* carrying the empty plasmid. Whereas, *Salmonella* carrying the empty plasmid was shown to limitedly delay tumor growth and hardly prolong the lifespan of mice if the treatment did not start early after tumor masses formed. As indicated by immunohistochemical studies, when mice were treated with endostatin delivered by our engineered *Salmonella*, the expression level of CD34 inside tumor tissue was significantly decreased compared to PBS or *Salmonella* treatment groups, thus confirming the *in vivo* anti-angiogenic activity of endostatin. *Salmonella* expressing TRAIL was also shown to cause increased cell apoptosis in tumor tissue, through staining for activated Caspase-3. Consistent with the overall anti-tumor effects observed, co-delivery of TRAIL and endostatin linked with RGD4C exhibited the strongest angiogenesis suppression and cell apoptosis, which was probably attributed to the two anti-tumor molecules delivered and *Salmonella* vector itself. The native anti-tumor activities of *Salmonella* targeting angiogenesis and cancer cells were also demonstrated through IHC studies.

We also noticed that the anti-tumor effects elicited by engineered *Salmonella* were accompanied by obvious toxicity to mice. After intraperitoneal infection of *Salmonella*, body weight of mice was decreased and gradually recovered to the normal level. *Salmonella* infection also caused the splenomegaly and pathological changes of normal tissues including liver and spleen. This reminds us that our engineered *S.* Typhimurium strain for delivery of anti-tumor molecules might not be attenuated enough. Systemic infection of *Salmonella* and subsequent bacterial accumulation inside the tumor could be achieved *via* the intraperitoneal route, while notable amounts of *Salmonella* were still able to colonize and persist in healthy tissues. Thus, the two auxotrophic mutations ∆*aroA* ∆*purM* are not sufficient for restricting *Salmonella* at the tumor site, and the hexa-acylated lipid A structure generated by Δ*pagP* Δ*pagL* Δ*lpxR* mutations may not be optimal for cancer therapy. Lipid A with further modifications, such as monophosphoryl lipid A, which has been clinically used as an adjuvant to enhance vaccine efficacy, should be investigated in future (Reed et al., 2009; Kong et al., 2011a,b, 2012). Other mutations are also required to further decrease bacterial colonization in healthy tissues as well, for the safety of clinical use. High throughput screening has advantages on generating *Salmonella* strains with reduced fitness in normal tissues but unaltered fitness in the tumor from thousands of candidate transposon-insertion mutants (Arrach et al., 2010). Re-isolation of bacteria from tumor tissue after *in vivo* infection seems to be another approach to further improve the tumor-targeting of *Salmonella* (Zhao et al., 2005, 2006). Recently, intratumoral infection of *Salmonella* has been shown to cause similar anti-tumor therapeutic benefits and reduced toxic-side effects when compared to intravenous administration. This route allows extensive dose of bacteria to bypass the killing by innate immunity encountered after systemic infection and to directly target cancer cells within tumor tissue (Hong et al., 2013; Kocijancic et al., 2017). However, it is not applicable to cancer patients with hard-to-reach tumors or highly dispersed metastasis.

In summary, in this study, different wild type *S.* Typhimurium bacterial strains were compared in their *in vitro* phenotypes and *in vivo* virulence, colonization, and native anti-tumor efficacy. The results demonstrated that the avirulent strain LT-2 may be not suitable for cancer therapy and that the three highly virulent strains 14028, SL1344, and UK-1 were similar in many bacterial properties. An attenuated *S.* Typhimurium mutant strain with the auxotrophic characteristic and hexa-acylated lipid A was generated from UK-1 without any genetic scars in the genome and then used for delivery of anti-tumor molecules. As a result, the live attenuated *Salmonella* bacterial vector and its delivered anti-angiogenic endostatin and apoptosis-inducing TRAIL conferred superior anti-tumor effects in mouse tumor models, although obvious toxic-side effects were also caused meanwhile. Future studies will aim to optimize *Salmonella* to maximize its accumulation at the tumor site while minimize its toxicity to normal tissues of the host by introducing other mutations into *Salmonella* or attempting other administration routes for *Salmonella* infection. The delivery of anti-tumor molecules will also be optimized to restrict drug action on cancer cells or their supporting cells, sparing normal cells.

## Data Availability Statement

The original contributions presented in the study are included in the article/Supplementary Material, further inquiries can be directed to the corresponding author.

## Ethics Statement

The animal study was reviewed and approved by Southwest University.

## Author Contributions

KL and QK initiated the research. KL led the design of *in vitro* and *in vivo* experiments, data acquisition and analysis, and manuscript preparation. RZ, HL, JZ, ZT, XZ, YZ, and MA aided in data acquisition. QK participated in experimental design, data analysis, and manuscript preparation and revision. All authors contributed to the article and approved the submitted version.

## Funding

This work was funded by the National Natural Science Foundation of China 31970874, the Chongqing Postdoctoral Science Foundation cstc2020jcyj-bshX0112, and the China Postdoctoral Foundation 2020M683217.

## Conflict of Interest

The authors declare that the research was conducted in the absence of any commercial or financial relationships that could be construed as a potential conflict of interest.

## Publisher’s Note

All claims expressed in this article are solely those of the authors and do not necessarily represent those of their affiliated organizations, or those of the publisher, the editors and the reviewers. Any product that may be evaluated in this article, or claim that may be made by its manufacturer, is not guaranteed or endorsed by the publisher.
