# Supplementary material for: Optimized Attenuated Salmonella Typhimurium Suppressed Tumor Growth and Improved Survival in Mice
Source: Front Microbiol. 2021 Dec 23;12:774490. doi: 10.3389/fmicb.2021.774490 (PMC8733734; doi:10.3389/fmicb.2021.774490)
Supplement: Supplementary file 1 [file Data_Sheet_1.docx]

# *Supplementary materials*

## Table S1. The primers used for construction of suicide and expression plasmids.

|  | **Primers** | **Sequences (5’→3’)** | **Used for** |
| --- | --- | --- | --- |
| 1 | 112-F | accctgataaatgcttcaataatgg | Cloning the backbone of PRE112 suicide plasmid |
| 2 | 112-R | gtcaacagctcatttcagaatgg |  |
| 3 | D*purM*-1F | ccattctgaaatgagctgttgacctctgacgcaacacaatgtatgc | Cloning upstream and downstream homologous arms of *purM* |
| 4 | D*purM*-1R | gaatcagaggtgcctggttccccacgttttttc |  |
| 5 | D*purM*-2F | gaaccaggcacctctgattccgaacagcgt |  |
| 6 | D*purM*-2R | ccattattgaagcatttatcagggtgaagggtgaatgttcagcagacg |  |
| 7 | pYA4088-F | taagcttggctgttttggcgg | Cloning the plasmid backbone with *bla* ss under Ptrc for expression |
| 8 | pYA4088-R | gaattcttcagcatcttttac |  |
| 9 | ES-F for 4088 *bla* | gaaagtaaaagatgctgaagaattccatagccatcgcgattttcaacc | Cloning *endostatin* with C-terminal his-tag |
| 10 | ES-his-R for 4088 | ccgccaaaacagccaagcttagtgatgatggtggtgatgtttgctcgcggtcataaagc |  |
| 11 | PSMA scFv-F for 4088 | gaaagtaaaagatgctgaagaattcgaggttcagctgcagcaaag | Cloning the cDNA encoding PSMA scFv and *endostatin* with C-terminal his-tag, and fusing them by CH1-linker |
| 12 | PSMA-R for CH1-linker | cggttccagcggataaacgctcggcgcggtggttttcgcacgtttaatttccagcttgg |  |
| 13 | ES-F for CH1-linker | cgtttatccgctggaaccggttagcagcggtagcggtcatagccatcgcgattttcaac |  |
| 14 | his tag-R for 4088 | atccgccaaaacagccaagcttagtgatgatggtggtgatg |  |
| 15 | CH1-F for RGD4C | gccgtggcgattgcttctgcggcgcgaaaaccaccgcgccgagc | Cloning CH1-linker for fusing RGD4C/RGD10 and *endostatin* |
| 16 | CH1-F for RGD10 | ctgccgtggcgattgcttcgacggcgcgaaaaccaccgcgccgagc |  |
| 17 | CH1 linker-R | accgctaccgctgctaaccggttc |  |
| 18 | new ES-F for CH1 linker | gaaccggttagcagcggtagcggtcatagccatcgcgattttcaac | Cloning the backbone of pYA3342-ES |
| 19 | 88 bla-R for RGD4C | gcagaagcaatcgccacggcagtcgcacgcgaattcttcagcatcttttac |  |
| 20 | 88 bla-R for RGD10 | gtcgaagcaatcgccacggcagtaacgcgcaccatcgaattcttcagcatcttttac |  |
| 21 | SD-*asd*-F | ggaaggtcttccggaagatccg | Cloning *asd* and the plasmid backbone of PET28 |
| 22 | *asd*-R | cttggtctgacagtctagactag |  |
| 23 | PET-F for *asd* | cggatcttccggaagaccttcccatgagcggatacatatttgaatg |  |
| 24 | PET-R for *asd* | ctagtctagactgtcagaccaagcaccccttgtattactgtttatg |  |
| 25 | *araC* ups-F for PET-*lacI* | tggcgcaaaacgaagcggcatgcataatgtg | Cloning *araC* P_BAD_ and the backbone of plasmid PET28-*asd*, of which *kanR* gene was replaced by *asd* |
| 26 | P_BAD_-R for PET-*lacI* | ctggtttcacggttaattcctcctgttagc |  |
| 27 | PET-F (*lacI*) for P_BAD_ | ggaggaattaaccgtgaaaccagtaacgttatacg |  |
| 28 | PET-R for *araC* | gcatgccgcttcgttttgcgccattcgatggtg |  |
| 29 | lac UV5-F for PET | ctatatcgccgacatcaccgatggctttacactttatgcttccg | Cloning gene encoding T7 RNA polymerase and the backbone of plasmid PET28-*asd*-P_BAD_ *lacI*, of which *lacI* was regulated by P_BAD_ promoter |
| 30 | BL21 T7 pol-R | gatccggagtcgtattgatttgg |  |
| 31 | PET-F for T7 pol | atcggtgatgtcggcgatatag |  |
| 32 | PET-R for T7 pol | ccaaatcaatacgactccggatcctcgccacttcgggctcatg |  |
| 33 | TRAIL-F | atgccttcttcaggcgcgctg | Cloning *TRAIL* and the backbone of plasmid PET28-*asd*-P_BAD_ *lacI*-*T7 pol*, into which *T7 pol* was inserted |
| 34 | TRAIL-R | gttaatcagaaacgcgccaaag |  |
| 35 | PET28a-F for TRAIL | cagcgcgcctgaagaaggcatggtatatctccttcttaaag |  |
| 36 | PET-R for TRAIL | ctttggcgcgtttctgattaaccaccaccaccaccaccactgagatc |  |
| 37 | Ptrc ups-F | ggatcttccggaagaccttcc | Cloning Ptrc-T1T2 ORF from pYA3342-derived expression plasmids and the backbone of plasmid PET28-*asd*-P_BAD_ *lacI*-*T7 pol*-*TRAIL*, where *TRAIL* was regulated by P_T7_ |
| 38 | T1T2 downs-R | gagtttgtagaaacgcaaaaagg |  |
| 39 | PET-F for T1T2 | cctttttgcgtttctacaaactccatgagcggatacatatttgaatg |  |
| 40 | PET-R for Ptrc | ggaaggtcttccggaagatccg |  |


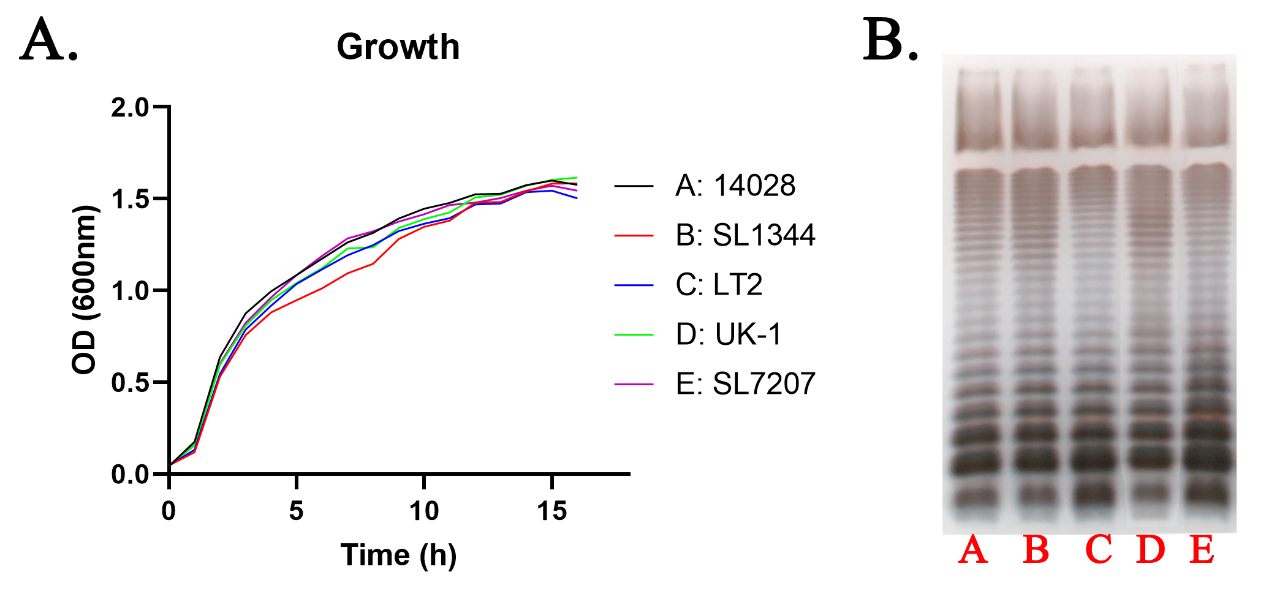


**Fig. S1. The growth and LPS profiles of different *S.* Typhimurium strains.**

(A) The growth of different *S.* Typhimurium bacterial strains was tested in LB medium (37 °C, 180 rpm). The OD_600_ values of bacterial cultures were measured every hour till the stationary phase of growth. (B) The LPS profile of bacterial strains were identified by silver staining.


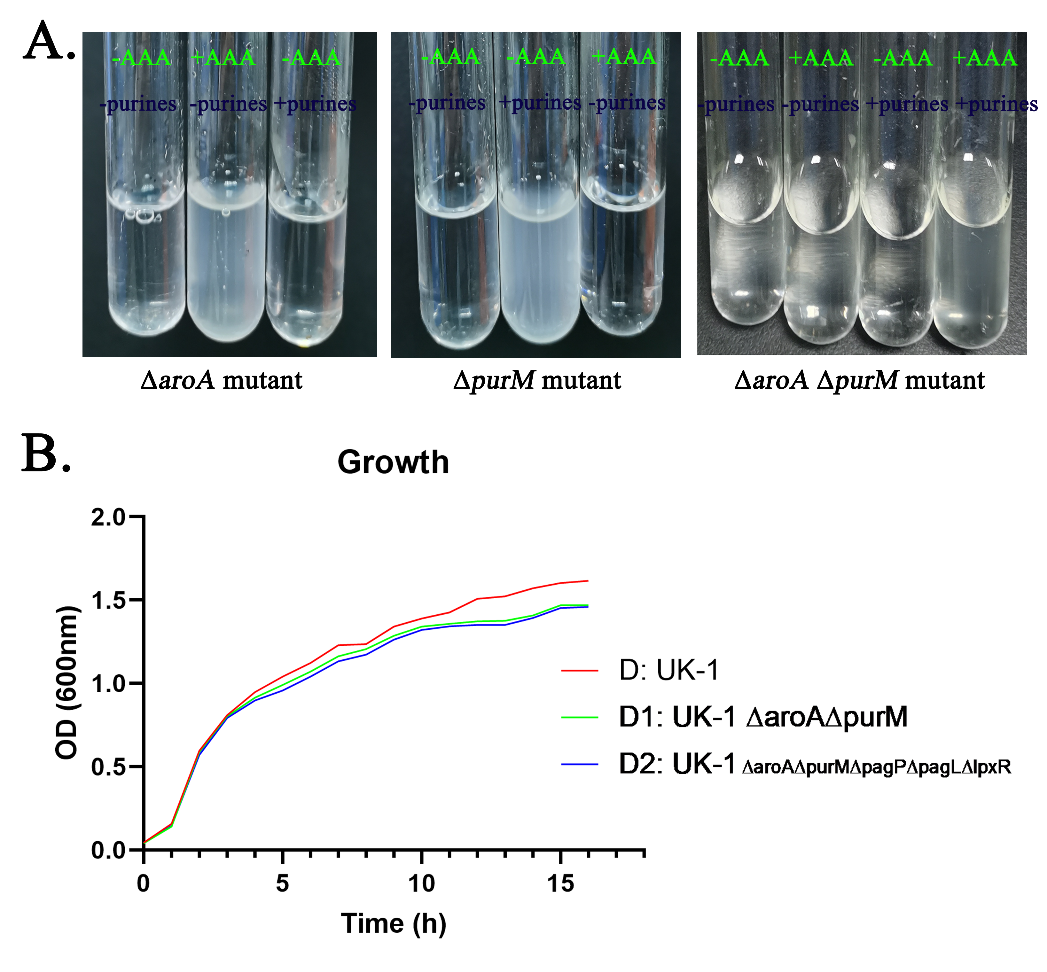


**Fig. S2. The auxotrophic phenotype of *S.* Typhimurium ∆*aroA*∆*purM* mutant strains.**

(A) The purine (adenine) and aromatic amino acids (tyrosine, phenylalanine and tryptophan) were added to minimal salt medium for testing the auxotrophic phenotype of ∆*aroA* and/or ∆*purM* mutant strains. (B) The growth curves of auxotrophic bacterial strains D1 and D2 as well as their wild type strain in LB broth was also measured.


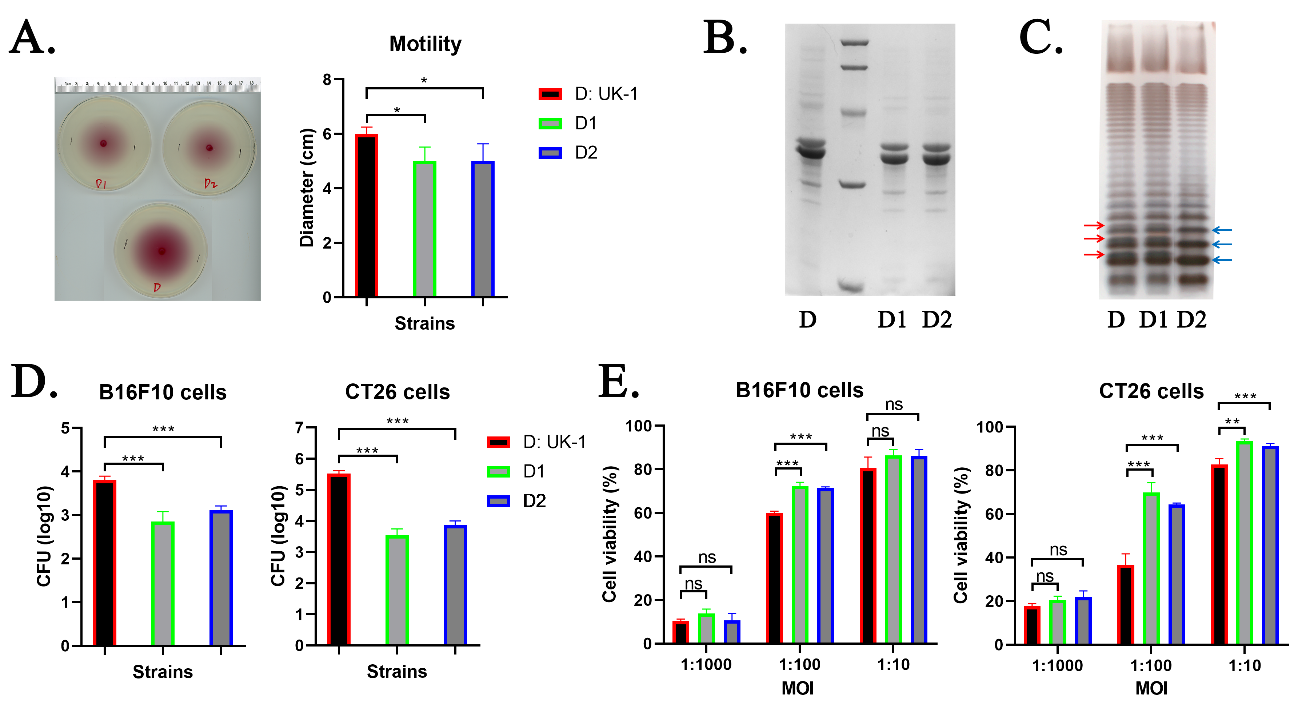


**Fig. S3. The *in vitro* phenotypes of engineered mutant strains derived from UK-1.**

(A) Swarming motility of bacterial strains was tested on 0.3% semi-solid agar plates and the diameter of swarming was measured with a ruler. (B) Outer membrane proteins purified from D (UK-1) and its derivatives D1 (∆*aroA* ∆*purM*) and D2 (∆*aroA* ∆*purM ∆pagP∆pagL∆lpxR*) were subjected to SDS-PAGE and stained by coomassie brilliant blue. (C) The LPS profiles of bacterial strains were identified by silver staining. Compared to D2 whose LPS are homogeneous (blue arrows), both the wild type strain D and the auxotrophic strain D1 can also synthesize LPS that migrate more slowly (red arrows), with the same number of O antigen units connected. (D) The ability of auxotrophic strains D1 and D2 to invade cancer cells was tested by counting as described and compared with that of UK-1. (E) The toxicity of bacterial stains to cancer cells of CT26 and B16F10 lines at different MOI was assessed by the CCK-8 assay. The significance of differences among groups were analyzed by two-way ANOVA analysis followed by Tukey's multiple comparisons test and indicated by asterisks (*, *P*< 0.05; **, *P*< 0.01; and ***, *P*< 0.001).


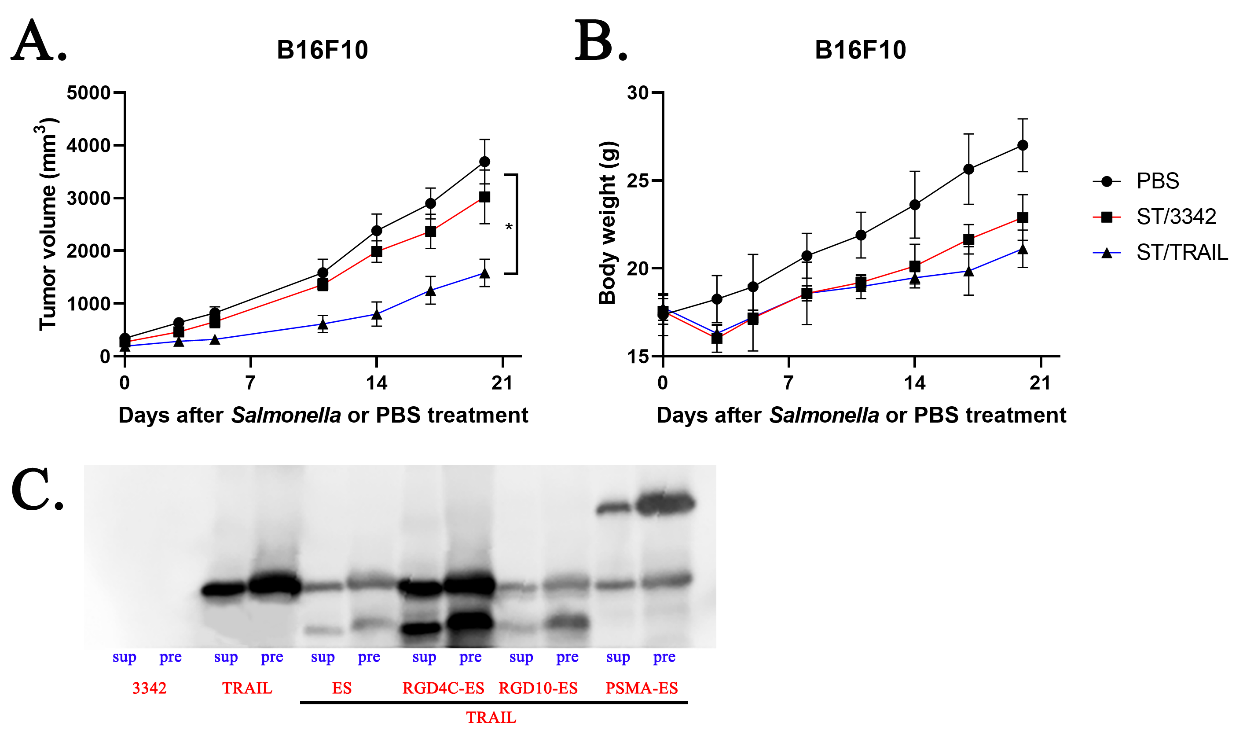


## Fig. S4. The *in vivo* anti-tumor efficacy of engineered TRAIL-expressing *Salmonella* and the *in vitro* co-expression and secretion of anti-tumor molecules by *Salmonella*.

Tumor-bearing mice were randomly divided into three groups and intraperitoneally injected with 100 μL of PBS or or 5 × 10^6^ CFU of attenuated *Salmonella* bacteria (D2-asd) carrying pYA3342 or plasmids expressing TRAIL. Tumor volume of melanoma-bearing mice (A) was measured every 2~3 days and meanwhile, body weight of mice (B) was also recorded as an indicator for general health status. (C) The co-expression and secretion of endostatin and TRAIL by *Salmonella* bacteria was confirmed by western blotting as described, before evaluation of the potential anti-tumor effects of such recombinant *Salmonella* bacteria.


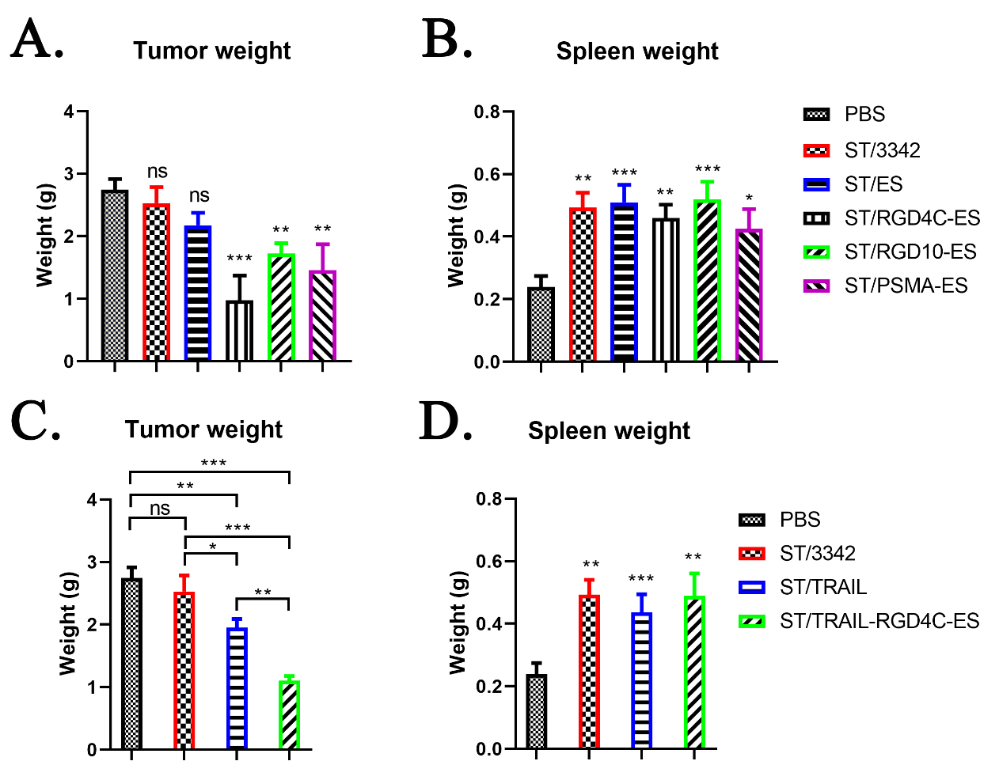


**Fig. S5. The weight of tumor and spleen tissues taken from tumor-bearing mice receiving different treatments.**

At dpi 14, mice bearing CT26 colon carcinoma from different groups were sacrificed and the tumor and spleen tissues were weighted. The weight was compared among groups through one-way ANOVA analysis**.**


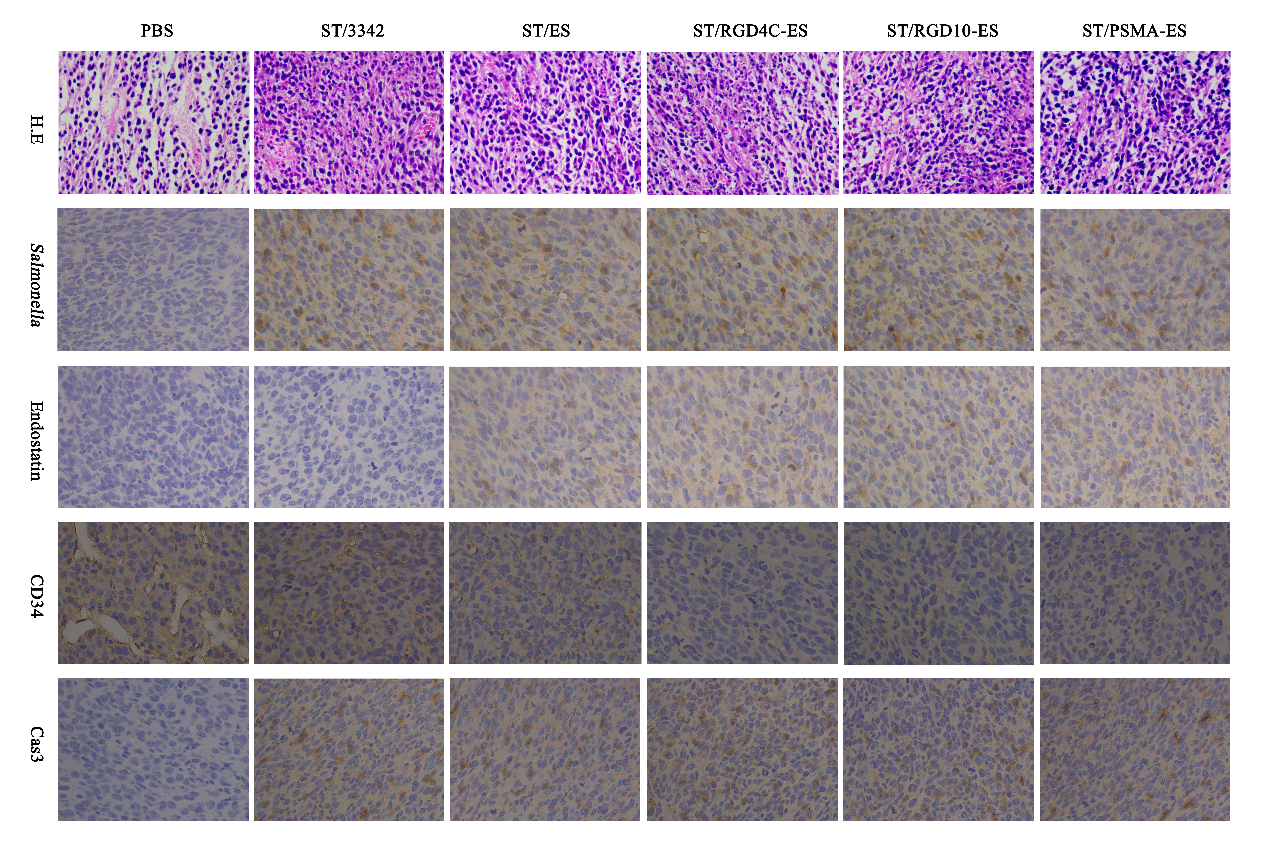


**Fig. S6. Immunohistochemical studies on tumor tissues infected by endostatin-expressing *Salmonella* bacteria.**

Two weeks after tumor-bearing mice received the treatment of PBS or attenuated *Salmonella* bacteria (D2-asd) carrying pYA3342 or plasmids expressing different forms of endostatin, mice were sacrificed and tumor samples were taken for IHC staining for *S.* Typhimurium O-antigen, endostatin, TRAIL, CD34 and activated caspase-3.


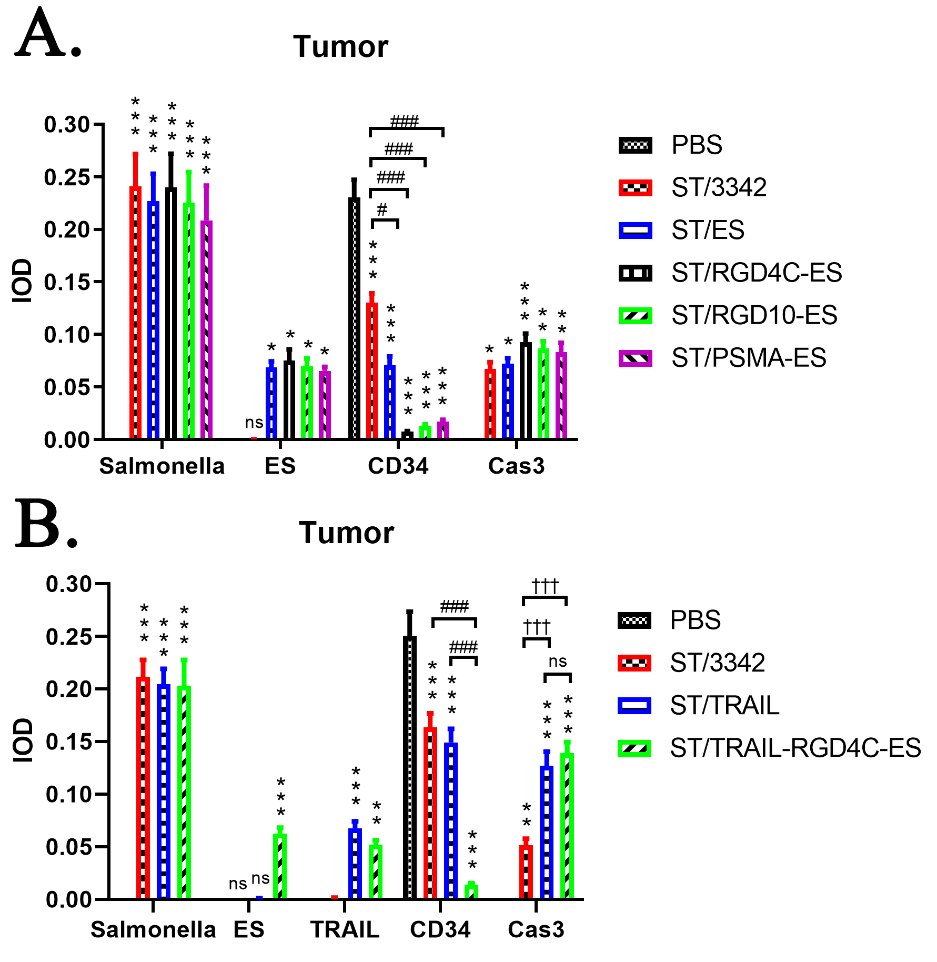


**Fig. S7. Analysis of immunohistochemical staining on tumor tissues.**

The positive IHC staining on tumor samples was semi-quantitatively analyzed by the software Image-Pro Plus 6.0 and indicated by mean IOD value. In this study, 3 tumors from each group and 5 fields of view from each tumor sections were taken for analysis. The significance of differences among groups were analyzed by one-way ANOVA test and indicated by asterisks (*, #, or †, P＜0.05; **, ##, or ††, P＜0.01; and ***, ###, or †††, P＜0.001).
